# An improved crystal structure of C-phycoerythrin from the marine cyanobacterium *Phormidium* sp. A09DM

**DOI:** 10.1007/s11120-017-0443-2

**Published:** 2017-09-16

**Authors:** Ravi R. Sonani, Aleksander W. Roszak, Claire Ortmann de Percin Northumberland, Datta Madamwar, Richard J. Cogdell

**Affiliations:** 1 Post-Graduate Department of Biosciences, UGC-Centre of Advanced Study, Sardar Patel University, Vadtal Road, Satellite Campus, Bakrol, Anand, Gujarat 388315 India; 2 Institute of Molecular Cell an Systems Biology, University of Glasgow, 120 University Place, Glasgow, G12 8TA UK

**Keywords:** Photosynthesis, Cyanobacteria, Phycobilisomes, Phycoerythrin, PEB chromophores, Atomic resolution crystal structure

## Abstract

C-Phycoerythrin (PE) from *Phormidium* sp. A09DM has been crystallized using different conditions and its structure determined to atomic resolution (1.14 Å). In order for the pigment present, phycoerythrobilin (PEB), to function as an efficient light-harvesting molecule it must be held rigidly (Kupka and Scheer in Biochim Biophys Acta 1777:94–103, 2008) and, moreover, the different PEB molecules in PE must be arranged, relative to each other, so as to promote efficient energy transfer between them. This improved structure has allowed us to define in great detail the structure of the PEBs and their binding sites. These precise structural details will facilitate theoretical calculations of each PEB’s spectroscopic properties. It was possible, however, to suggest a model for which chromophores contribute to the different regions of absorption spectrum and propose a tentative scheme for energy transfer. We show that some subtle differences in one of these PEB binding sites in two of the 12 subunits are caused by crystal contacts between neighboring hexamers in the crystal lattice. This explains some of the differences seen in previous lower resolution structures determined at two different pH values (Kumar et al. in Photosyn Res 129:17–28, 2016).

**Electronic supplementary material:**

The online version of this article (doi:10.1007/s11120-017-0443-2) contains supplementary material, which is available to authorized users.

## Introduction

The major light-harvesting complexes of cyanobacteria and red algae are the phycobilisomes (Zhao et al. 2012). These large, water-soluble pigment–protein complexes bind to the surface of the photosynthetic membranes and funnel absorbed light energy into the chlorophyll-containing photosystems. The phycobilisomes are usually made up of phycoerythrin (PE), phycocyanin (PC) and allophycocyanin (APC). They are arranged so that PE (absorption maximum A_max_ 540–570 nm) is on the outside of the structure, PC (A_max_ 610–620 nm) is in the middle and APC (A_max_ 655 nm) is nearest to the photosynthetic membrane. This then sets up an energy gradient that ‘directs’ energy transfer downhill to the photosystems. PE and PC typically form rods that join onto the central APC discs. Mainly colorless linker proteins play a key role in organizing the overall structure of the phycobilisome and subtly modulate the absorption properties of the phycobiliproteins to which they bind.

**Fig. 1:**
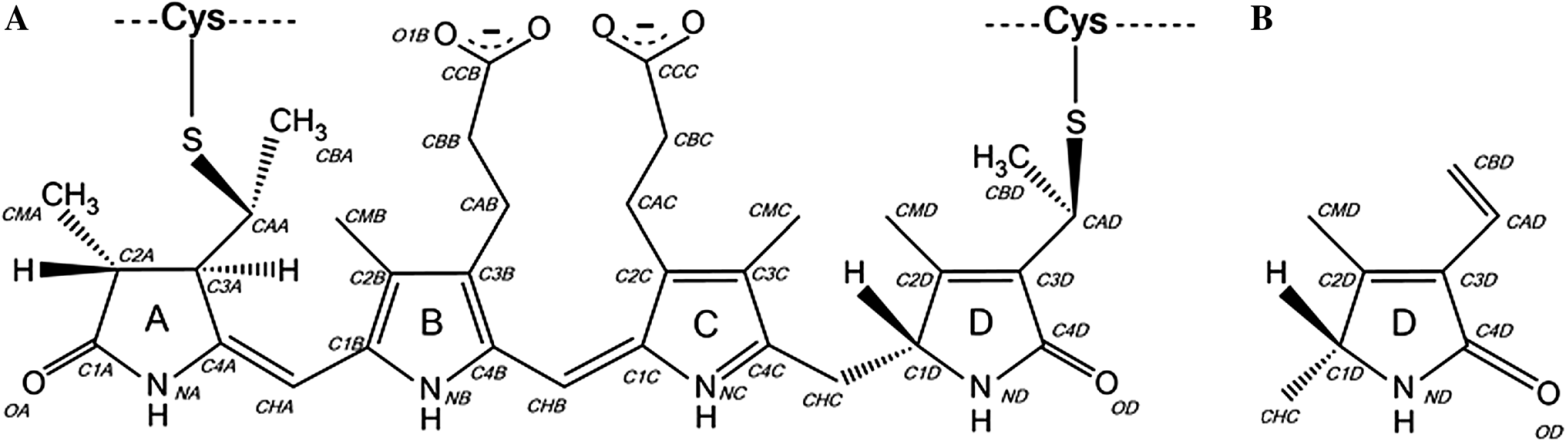
**a** Chemical structure of phycoerythrobilin PEB188, which is attached to PE protein via two thioether bonds; **b** chemical structure of D-ring of the PEB chromophore attached to protein by a single thioether bond; rings A, B and C are identical as in PEB in diagram (**a**)

PEs are oligomers of a basic heterodimer that consists of α- and β-subunits (conventionally referred as αβ monomer) to which bile pigments are covalently attached through conserved cysteine residues. These monomer units then oligomerise to form trimers [(αβ)_3_] and then stacked hexamers {[(αβ)_3_]_2_}. The structure of this complex was first determined at two different pH values of 5 and 8.5 to the resolutions of 1.93 and 2.12 Å, respectively (PDB IDs: 5fvb and 5aqd, respectively; Kumar et al. 2016). Since then we have been able to significantly improve the quality of the crystals of this pigment–protein complex and have determined its structure to atomic resolution of 1.14 Å. The best possible structures for light-harvesting complexes are important since this information can then be used for theoretical studies designed to understand the detailed spectroscopic properties of the pigments involved in the light-harvesting processes. For example, see the quantum mechanics/molecular mechanics calculations performed for the cryptophyte PE545 antenna (Aghtar et al. 2017; Curutchet and Mennucci 2017 and other references within) based on the 0.97 Å resolution structure of PE545 from unicellular cryptophyte *Rhodomonas CS24* (Doust et al. 2004). In the case of PE from *Phormidium* sp. A09DM the pigment present is phycoerythrobilin (PEB) bound covalently to PE protein via one or two C_PEB_–S_Cys_ bonds; its structure is shown in Fig. 1. One of the major ways in which the apoprotein controls where an individual PEB absorbs (its site energy) is by ‘distorting’ the planarity of the bilin. This then changes the effective conjugation length (Gaigalas et al. 2006). Reducing the conjugation length will cause a blue shift of the PEB’s absorption spectrum. This effect was described previously (Doust et al. 2004; Curutchet et al. 2013) for the PEB pigments from cryptophyte PE545. On top of this the site energies of the individual PEB molecules will be further influenced by more subtle aspects of the pigments’ local environment (Doust et al. 2004; Curutchet et al. 2011; Aghtar et al. 2017). The present report will describe the overall PEB binding pockets in more detail than was previously discussed for the cyanobacterial PE from *Phormidium* sp. A09DM (Kumar et al. 2016). This earlier study also described subtle differences in the two crystal structures related to crystals being grown at different pHs, pH 5 and pH 8.5, respectively. Our atomic resolution structure explains these previous findings.

## Materials and methods

### Protein purification

The PE was isolated and purified from marine cyanobacteria *Phormidium* sp. A09DM as described earlier (Sonani et al. 2015; Kumar et al. 2016).

### Analysis of absorption spectrum

The absorption spectrum of PE in 20 mM Tris-buffer (pH 8.0) was recorded at 25 °C in a UV–visible spectrophotometer (Specord 210, Analytik Jena AG, Jena, Germany). This spectrum was then deconvoluted into Gaussian decomposition components using the Gaussian algorithm present in the OriginPro8 software (OriginLab, Northampton, MA).

### Circular dichroism (CD) spectrum

CD spectrum of PE in the region of 400–600 nm was recorded at room temperature in 0.2 cm cuvette using a J-810 spectrophotometer (JASCO). The PE sample was dissolved in 20 mM Tris-buffer (pH 8.0) and had an absorption of 1 at the maximum of the absorption spectrum.

### Crystallization of PE

Crystallization trials of purified PE were set up using the following range of sparse matrix screens, JCSG-plus, PACT premier, Morpheus, MIDAS and Structure, which were all obtained from Molecular Dimensions. The screening was carried out by a crystallization robot Cartesian Honeybee 8+1 (Genomic Solutions Ltd) using sitting drop vapour diffusion in 96-well 2-drop MRC crystallization plates (Molecular Dimensions). These trials (with PE at an OD 75 at 564 nm) yielded reasonable size crystals (100 × 80 × 80 microns) within 6 days in conditions C8 [37.5% v/v Precipitant Mixture MPD_P1K_P3350; Buffer System 2 [Na HEPES; MOPS (acid)] pH 7.5; 0.09 M mix of additive nitrate phosphate sulphate (NPS)] and G8 [37.5% v/v Precipitant Mixture MPD_P1K_P3350; Buffer System 2, pH 7.5; 0.1 M mix of carboxylic acids] of the Morpheus screen. Both conditions at pH 7.5, C8 and G8, were further optimized using a larger volume 24-well plates (Cryschem, NBS Biologicals) and the purified PE in a buffer consisting of 20 mM Tris-HCl pH 8.0, concentrated to 10 mg/mL. Diffraction-quality crystals of 300 × 200 × 100 micron were obtained in 15 days in both conditions. The details of the optimized conditions are shown in Table S1 in the Supplementary Material.

### Data collection and analysis

The optimized PE crystals were scooped up from the crystallization droplets in the LithoLoops (Molecular Dimensions) and flash-cooled in nitrogen gas at 100 K. Both Morpheus crystallization conditions, C8 and G8, contain sufficient amounts of cryoprotectants like MPD and PEG-1K so no extra cryoprotection was needed. These crystals diffracted to about 1.5–1.7 Å resolution during tests using our in-house X-ray setup (Rigaku MicroMax-007 generator and *mar*345*dtb* detector). The best crystals were sent to the Diamond Light Source (DLS) synchrotron near Oxford, UK, where diffraction data were collected at 100K on beamlines I04 and I04-1 using detector Pilatus 6M-F (Dectris AG). Table 1 presents the data collection, processing and refinement statistics for the highest-resolution datasets obtained for both pH 7.5 crystals, from Morpheus C8 and G8 conditions. The data were indexed, integrated, scaled and evaluated using the following programs; XDS (Kabsch 2010), POINTLEES (Evans 2006), AIMLESS (Evans and Murshudov 2013), autoPROC (Vonrhein et al. 2011), *xia2* (Winter 2010) and the other programs from the CCP4 suite (Winn et al. 2011).

**Table 1:** Data collection, processing and refinement statistics for the PE complex from *Phormidium* sp. A09DM

Morpheus crystallization condition	C8	G8
Protein Data Bank accession code	5nb4	5nb3
Space group	*P1*	*P1*
Unit cell *a, b, c* (Å)	109.046, 109.095, 117.371	110.046, 110.170, 118.516
Unit cell *α, β, γ* (°)	78.78, 82.32, 60.26	78.76, 82.28, 60.43
Data measured at beamline	I04 at DLS	I04-1 at DLS
Detector used	Pilatus 6M-F	Pilatus 6M-F
Wavelength (Å)	0.97951	0.92819
Resolution range (outer shell)^a^ (Å)	94.60–1.14 (1.16–1.14)	93.40–1.38 (1.42–1.38)
Unique reflections	1,575,226 (74,960)	932,250 (67,249)
Redundancy	2.6 (2.7)	2.9 (2.8)
Completeness (%)	93.8 (90.0)	95.7 (93.2)
*R*_merge_^b^ (%)	4.2 (54.5)	7.7 (80.1)
Mean *I*/σ	10.7 (1.6)	7.3 (1.3)
Half-set correlation coefficient^c^ CC_1/2_	0.998 (0.601)	0.997 (0.477)
Refinement *R*_work_/*R*_free_ factors^d^ (%)	14.7/18.4	15.5/21.1
Ramachandran plot features^e^ (%)	98.2/1.5/0.3	98.1/1.6/0.3
Rms dev. bond lengths/angles (Å/°)	0.017/2.68	0.018/2.60
Coordinate error^f^ (Å)	0.035/0.031	0.066/0.059
No. of non-H atoms used in refinement	43,607	43,181
No. of water molecules	8168	7843
Mean atomic/Wilson plot *B* factors (Å^2^)	15.6/9.7	15.9/8.2

^a^Values in parentheses are for the highest-resolution outer shell

^b^ $${R_{{\text{merge}}}}={{{\sum _{hkl}}{\sum _i}\left| {{I_i}\left( {hkl} \right) - <I\left( {hkl} \right)>} \right|} \mathord{\left/ {\vphantom {{{\sum _{hkl}}{\sum _i}\left| {{I_i}\left( {hkl} \right) - <I\left( {hkl} \right)>} \right|} {{\sum _{hkl}}{\sum _i}{I_i}\left( {hkl} \right)}}} \right. \kern-0pt} {{\sum _{hkl}}{\sum _i}{I_i}\left( {hkl} \right)}}$$

^c^The experimental unmerged data are divided into two parts, each containing a random half of the measurements of each unique reflection. The correlation coefficient CC_1/2_ is then calculated between the average intensities of each subset (Karplus and Diederichs 2012)

^d^ ${R_{{\text{work}}}}\;\text{and}\;{R_{{\text{free}}}}={{{\sum _{hkl}}\left| {\left| {{F_o}\left( {hkl} \right)} \right| - \left| {{F_c}\left( {hkl} \right)} \right|} \right|} \mathord{\left/ {\vphantom {{{\sum _{hkl}}\left| {\left| {{F_o}\left( {hkl} \right)} \right| - \left| {{F_c}\left( {hkl} \right)} \right|} \right|} {{\sum _{hkl}}\left| {{F_o}\left( {hkl} \right)} \right|}}} \right. \kern-0pt} {{\sum _{hkl}}\left| {{F_o}\left( {hkl} \right)} \right|}}$; *R*_work_ was calculated for all data except for 5% that was used for the *R*_free_ calculations

^e^Percentages of residues in most favoured/additionally allowed/disallowed regions by RAMPAGE (Lovell et al. 2003)

^f^Estimated standard uncertainty; first value calculated using the method of Cruickshank (Cruickshank 1999), second one based on maximum likelihood as implemented in REFMAC (Murshudov et al. 2011)

The symmetry of data and of the pH 7.5 crystals was found to be *P*1 as was the symmetry of the pH 5 and 8.5 crystals obtained before (Kumar et al. 2016). Similarly, the unit cell dimensions of all crystals are almost identical with no bigger difference than 1.7%. The asymmetric unit of these crystals, the whole unit cell, is comprised of 12 αβ monomers organized in two stacked hexamers {[(αβ)_3_]_2_}. The 12 α-subunits are labelled A to L, while the 12 β-subunits are labelled M to X. There are two PEB molecules, PEB166α and PEB167α, attached to each α-subunit and three, PEB186β, PEB187β and PEB188β, to each β-subunit.

Due to high internal similarity of the new crystals to the earlier ones the phasing of diffraction data was directly obtained by the process of rigid-body refinement in REFMAC5 (Murshudov et al. 2011) of the 2.12 Å pH 8.5 structure of PE from *Phormidium* sp. A09DM (PDB ID: 5aqd; Kumar et al. 2016) against the new 1.14 Å data. The *R*_free_ reflections were also copied from the 5aqd structure and extended to 1.14 Å resolution, then copied again to 1.38 Å data. Model refined against 1.14 Å data was then used and remodelled when necessary in refinement against the 1.38 Å data. All structure modelling was performed in COOT (Emsley et al. 2010). Due to the available amount of the experimental data the refinements of both pH 7.5 PE structures presented here were finished with the introduction of anisotropic atomic B factors. Program RAMPAGE (Lovell et al. 2003) was used to evaluate stereochemistry of the structures and only the single residue in β-subunit, Pro73, was found slightly off the allowed region of the Ramachandran plot in both structures, although it was perfectly fitted within the strong electron density. The atomic coordinates and structure factors have been deposited in the Protein Data Bank with the accession ID codes 5nb4 and 5nb3, for the PE from Morpheus conditions C8 and G8, respectively.

## Results and discussion

### Atomic resolution structure of C-phycoerythrin

PE from *Phormidium* sp. A09DM has been successfully crystallized from two different Morpheus screen conditions at the pH of 7.5. Details of the general composition of this PE protein and its crystal structure have been reported earlier (Kumar et al. 2016). The description of the atomic resolution structure of PE will be presented here using the model based on the 1.14 Å data for the crystal grown in NPS conditions (Morpheus C8) while the model based on the 1.38 Å data (crystal grown in carboxylic acids mix, Morpheus G8) will only be mentioned explicitly in the cases of significant structural differences.

**Fig. 2:**
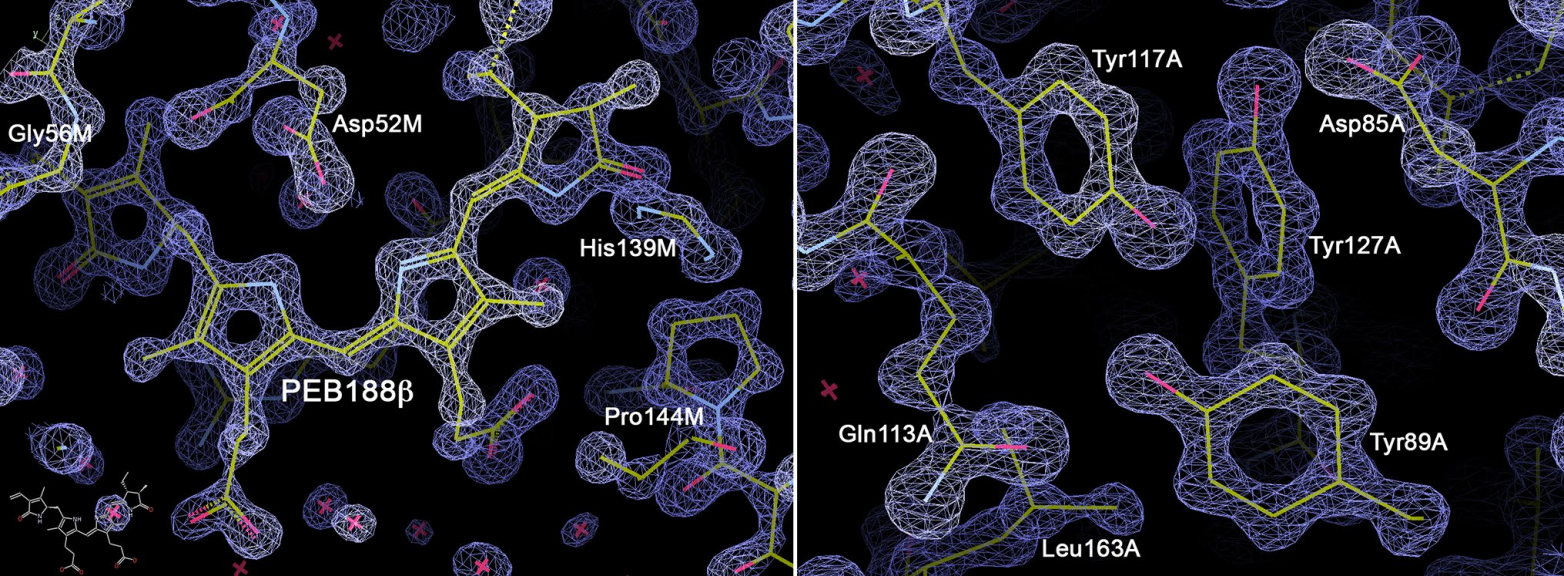
Representative examples of the 1.14 Å electron density map *2Fo-Fc* at contour level 2.5 sigma for the PE from *Phormidium* sp. A09DM; these images were prepared using program COOT (Emsley et al. 2010)

Experimental data extending to 1.14 Å resolution allow for a very high precision of structure to be determined. The coordinate error estimated in the maximum likelihood refinement of this structure is 0.03 Å, which is almost an order of magnitude smaller than the equivalent value (0.25 Å) estimated for the earlier 1.93 Å pH 5 model (PDB ID: 5fvb; Kumar et al. 2016). This difference in precision is important for the quality of structure-based theoretical modelling of spectroscopic properties (Mennucci 2017, personal communication). Figure 2 illustrates the quality of electron density maps calculated for the 1.14 Å resolution PE structure. A typical refinement of the protein structure model is performed by the use of the combination of experimental X-ray diffraction intensities produced by the protein crystal and the restraints coming from the prior knowledge of protein structure bond distances, bond angles and torsion angles. The higher the resolution is, and therefore the number of the experimental data, the larger is the contribution of the actual X-ray diffraction experiment in such a refinement. At resolutions better than about 1.2 Å (Rupp 2010) discrete atoms are distinctly visible in the electron density maps and these effects are clearly seen in Fig. 2.

### Description of protein microenvironments of chromophores

Deviations from planarity of the five PEB pigment molecules present in each PE αβ monomer and the likely effects of these on the position of the pigment’s absorption maxima were already described earlier (Kumar et al. 2016). These deviations are represented by the angles between the pyrrole rings A and B, which are part of the conjugated A–B–C-ring system of PEB pigment (see Fig. 1). The B- and C-rings are approximately coplanar with each other in all PEB molecules found in the PE structures, while the ring A strongly deviates from this central ‘B–C’ plane. It has been shown that such deviations from coplanarity of the A- and B-rings correlate with a decrease of the extent of π-coupling of the PEB-conjugated system and cause a blue shift in the position of the absorption maximum (Gaigalas et al. 2006). The average values of these deviations for the 12 independent molecules in the *P*1 cell for the crystal diffracting to 1.14 Å resolution are 25.6(1.0)°, 39.4(2.3)°, 21.5(0.9)°, 32.8(1.2)° and 42.6(1.0)° for the chromophores PEB166α, PEB167α, PEB186β, PEB187β and PEB188β, respectively. Values in brackets are the standard deviations for the 12 molecules in the cell. The exact angles for the individual 60 pigments are shown in Table S2 in the Supplementary Material. The authors of the earlier work (Kumar et al. 2016) concluded that the direction of energy flow could, therefore, be expected to be from the PEB molecules in the outer surface of the hexamer {[(αβ)_3_]_2_}, which show ‘high-deviations’ from planarity and, consequently, an absorption at shorter wavelengths compared to those of the ‘lower-deviation’ pigments (absorbing in longer wavelengths) located near to hollow inner cavity. In vivo, this cavity harbours the linker polypeptide (also called γ-subunit).

**Fig. 3:**
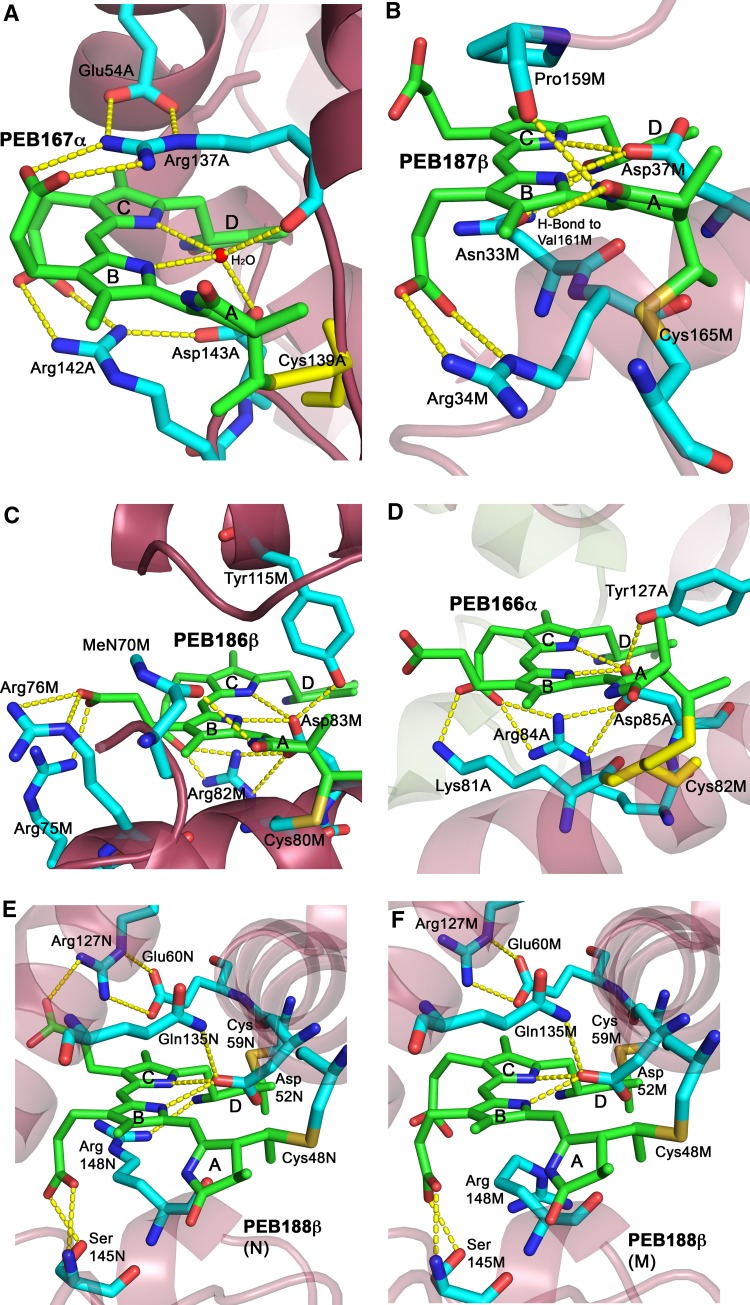
Geometry and interactions of chromophores **a** PEB167α in α-subunit A (same for B to L), **b** PEB187β in β-subunit M (same for N to X), **c** PEB186β in β-subunit M (same for N to X), **d** PEB166α in α-subunit A (same for B to L), and **e** PEB188β in β-subunit M (same for X), and **f** PEB188β in β-subunit N (same for O to W), within protein matrix. Hydrogen bonds are presented as yellow dashed lines. Water molecules are shown as red spheres. This figure was prepared using PyMOL (Schrodinger, LLC)

In PE from *Phormidium* sp. A09DM the PEB167α pigment is the outer-most ‘high-deviation’ chromophore, which is located on the outside surface of the α-subunit. The specific protein microenvironment of the PEB167α molecule, which is shown in Fig. 3a for the α-subunit A (the same arrangement is found for α-subunits B…L), compensates for its high exposure to the solvent phase and holds it in a well-defined fixed orientation. First of all the Asp143 and the carbonyl group of Arg137 residue anchor the PEB167α molecule in place via two H-bonds to a water molecule which itself forms two H-bonds to the NB and NC atoms of pyrrole rings B and C, respectively. Secondly, the side chains of two arginine residues, Arg137 and Arg142, interact via π-stacking with the central conjugated moiety of the PEB167α, hence contributing to a well-defined orientation of this pigment molecule. These arginine side chains are kept in place by forming two H-bonds each with the PEB B- and C-ring propionic groups, respectively. Additionally, residue Glu54 also interacts via two H-bonds with the Arg137 side chain, while the Asp143 helps to hold the Arg142 side chain via one more H-bond. Finally, the C_PEB_–S_Cys_ covalent bond between A-ring of PEB167α and Cys139, plus the H-bonds between the D-ring carbonyl group and residues Asn47 and Lys43 (not shown in Fig. 3a), hold the pigment rings A and D in place, respectively, and also fix the deviation of ring A from planarity from the central conjugated portion of PEB167α. Several more H-bonds between PEB167α and the surrounding solvent water molecules are formed, for full details of these interactions and also of all hydrophobic contacts see the LigPlot+ (Laskowski and Swindells 2011) diagram (Fig. S1A) in the Supplementary Material. In particular, one water molecule links the pigment’s B-ring propionic group with its A-ring NA atom while another one links the pigment’s C-ring propionic group with its D-ring ND atom, so they both also contribute to the control of the overall shape and rigidity of this pigment molecule.

PEB187β, the second ‘high-deviation’ chromophore, is located a bit deeper into the β-subunit of PE but is still partially exposed to the solvent phase outside of the hexamer. The protein microenvironment of this pigment is shown in Fig. 3b for β-subunit M (the same arrangement is found for β-subunits N…X). Residues Cys165 and Asp37 hold the PEB187β molecule in place via the C_PEB_–S_Cys_ covalent bond between Cys165 and PEB ring A, and the two H-bonds between the Asp37 side chain and the pigment’s NB and NC atoms of its rings B and C, respectively. Additionally, the two H-bonds between Arg34 side chain and PEB B-ring propionic group fix the defined orientation of the PEB187β molecule. The second PEB propionic group, bound to ring C, interacts by H-bonding with four water molecules, one of them H-bonds residue Gln147 from a neighboring α-subunit D (not shown in Fig. 3b, see full details in Fig. S1B in the Supplementary Material). Finally, beside the C_PEB_–S_Cys_ covalent bond there are also four H-bonds which hold the pigment rings A and D in place and fix the deviations of these rings from planarity relative to the central conjugated portion of PEB187β. The carbonyl O-atom of Pro159 and the main chain N-atom of Val161 (residue not shown, H-bond shown in Fig. 3b) are H-bonding the NA and carbonyl O-atoms of the pigment’s A-ring, respectively, while the side chains of Gln28 (from the α-subunit A, not shown in Fig. 3b) and Asn33 are H-bonding the D-ring carbonyl O- and NA atoms, respectively. In the PEB187β molecule there are no internal cross-links between the PEB A- and D-rings, and the propionic groups.

The chromophore PEB186β of β-subunit is located near the central hexamer cavity and it is one of the two pigments that show the least deviation from planarity (the other being PEB166α). The protein microenvironment of this pigment is shown in Fig. 3c for chain M (the same arrangement is found for β-subunits N…X). Residues Cys80 and Asp83 hold the PEB186β molecule in place via the C_PEB_–S_Cys_ covalent bond between Cys80 and PEB ring A, and the two H-bonds between the Asp83 side chain and the pigment’s NB and NC atoms of its rings B and C, respectively. The Asp83 side chain is held in place by the H-bond to Tyr115. The PEB186β molecule orientation is held by the PEB B- and C-ring propionic groups interacting via three H-bonds with side chains of residues Arg75 and Arg76 for B-ring and Arg82 for C-ring, respectively. Two propionic groups are also ‘linked’ by the H-bonds to a single water molecule (not shown in Fig. 3c). Finally, beside the C_PEB_–S_Cys_ covalent bond there is also one H-bond, between the ring’s NA atom and the side chain of residue MeN70 (methylated Asn, γ-*N*-methylasparagine), that holds the pigment ring A in place and helps to define the deviation of the ring A from planarity from the central conjugated rings B and C of the PEB186β. Depending on the β-subunit either two or three H-bonds are formed between the D-ring and solvent water molecules, of which one always H-bonds Arg82 side chain, and these are shown together with all hydrophobic interactions in Fig. S1C in the Supplementary Material.

The chromophore PEB166α is the second pigment that has a low deviation from planarity. It is located about halfway between the inner cavity and the outer-most perimeter of the {[(αβ)_3_]_2_} hexamer, and near the interface between α- and β-subunits of two different αβ monomers in the (αβ)_3_ trimer. In spite of being located near that interface the PEB166α pigment is completely embedded and only interacts with the α-subunit protein except for one water-mediated H-bonding contact with the neighboring β-subunit. The protein microenvironment of this pigment is shown in Fig. 3d for the α-subunit A (the same arrangement is found for α-subunits B…L). Residues Cys82 and Asp85 hold the PEB166α molecule in place via the C_PEB_–S_Cys_ covalent bond between Cys82 and PEB ring A, and the two H-bonds between the Asp85 side chain and the pigment’s NB and NC atoms of its rings B and C, respectively. The position of the Asp85 side chain is well fixed by the two H-bonds to Arg84 and by one to Tyr127. The orientation of the PEB166α is fixed by the PEB C-ring propionic group interacting via two H-bonds with side chains of residues Lys81 and Arg84, and the PEB B-ring propionic group making several H-bonds to water molecules, of which two also link via H-bonds to residues Lys81 and Leu120 (Leu120 not shown in Fig. 3d). The C-ring propionic group is also H-bonded to two water molecules, one of them linking the two propionic groups together (not shown in Fig. 3d), and another cross-linking to PEB D-ring’s NA atom. Finally, beside the C_PEB_–S_Cys_ covalent bond, there is also one H-bond between the NA atom of ring A and residue Ala72 (via main chain carbonyl bond, not shown), which holds the pigment ring A in place and adds to the stabilization of the deviation of the ring A from planarity from the central conjugated rings B and C of the PEB166α. The PEB D-ring interacts with the surrounding protein via an H-bond to a water molecule, which in turn H-bonds the carbonyl group of residue Cys71 from a neighboring β-subunit (not shown in Fig. 3d). Full details of the H-bonds to water molecules and of all hydrophobic interactions are shown in Fig. S1D in the Supplementary Material.

The fifth chromophore, PEB188β of the β-subunit, is also a ‘high-deviation’ chromophore located on the outer surface of the hexamer but its particular interactions with the protein, in the PE structure from *Phormidium* sp. A09DM reported here, are influenced by the 146–152 insertion (Kumar et al. 2016) in the neighboring protein loop and are, therefore, different from those seen in PE proteins without this insertion [see for instance B-phycoerythrin from *Porphyridium (P.) cruentum*, PDB ID: 3v57 (Camara-Artigas et al. 2012)]. The protein microenvironment of this pigment is shown in Fig. 3e, f for the β-subunits N and M, respectively. First of all, the PEB188β molecule is uniquely linked to the protein via two C_PEB_–S_Cys_ covalent bonds on the two ends of the pigment, to Cys48 at the A-ring end, and to Cys59 at the D-ring end. Additionally, two H-bonds are formed between the side chain of Asp52 and the pigment’s NB and NC atoms of its rings B and C, respectively, while the position of this Asp52 side chain is well fixed by the H-bond to residue Gln135 and the water-mediated H-bond to Arg148 main chain carbonyl group (this last interaction is not shown in Fig. 3e, f, full details of the PEB188β contacts with water molecules and of all hydrophobic interactions are shown in Fig. S1E, F in the Supplementary Material). All these interactions fix rigidly the position of the PEB188β molecule in the β-subunit and they are common for all the 12 β-subunits in the PE structure from *Phormidium* sp. A09DM. These interactions, except for the contact to Arg148, are also observed in PE structures without the 146–152 insertion such as PE from *P. cruentum* (Camara-Artigas et al. 2012). The PEB B-ring propionic group forms two H-bonds to residue Ser145 and one to a water molecule, which is also H-bonded to the A-ring NA atom of the PEB188β molecule. First two of these interactions help to fix the orientation of the PEB188β pigment, while the third one helps to define the deviation of the ring A from planarity relative to the central conjugated rings B and C of the PEB188β. All these interactions are common for all twelve β-subunits, and are similar to those found in PE from *P. cruentum* (Camara-Artigas et al. 2012) except that Ser145 is effectively replaced there by Thr147.

In contrast to the above discussion, the interactions of the PEB C-ring propionic group with the protein in the pH 7.5 structure of PE from *Phormidium* sp. A09DM reported here vary depending on which β-subunit is being discussed. The situation found in ten β-subunits, from N to W, is shown in Fig. 3e, where the PEB C-ring propionic group is H-bonded to the side chain of Arg127, the position of which is fixed by two H-bonds to the side chain of Glu60 (in two cases out of ten, in β-subunits P and S, this interaction is mediated by a water molecule). The side chain of Arg148, a residue from the 146–152 insertion, has a strong π-stacking interaction with the central conjugated portion of the PEB188β, in particular with its C-ring. This residue forms also an H-bond to an ND atom of the pyrrole ring D of PEB. A completely different situation was found in two remaining β-subunits, M and X, which is shown for β-subunit M in Fig. 3f. The PEB C-ring propionic group here turns away from the side chain of Arg127, while the side chain of Arg148 is bent and no longer involved in a π-stacking interaction with the conjugated PEB ring system. In their different conformations the C-ring propionic acid and the Arg148 in β-subunit M are linked by two H-bonds mediated by a water molecule (not shown if Fig. 3f, see Fig. S1F for details of all PEB interactions). Additionally, both PEB propionic groups and the side chain of Arg148 form several H-bonds mediated by water molecules to residues Thr122 and Thr123 from the β-subunit W of the neighboring hexamer in the crystal lattice. In case of β-subunit X, which is located on the second independent hexamer in this crystal structure, the Arg148 was fitted into the electron density with two alternative conformations of the side chain and these make H-bonds to C-ring propionic acid either directly or via a molecule of water. Again, both PEB propionic groups and the side chain of Arg148 form several H-bonds mediated by water molecules to residues Thr122 and Thr123 but this time from the β-subunit N of another neighboring hexamer in the crystal lattice. Finally, in the β-subunits N to W, the PEBs ring D beside being linked to protein by the C_PEB_–S_Cys_ covalent bond forms a water-mediated H-bond to residue Glu60 and another H-bond to π-stacked side chain of Arg148, of which the latter was mentioned already earlier. In the β-subunits M and X, the PEBs ring D beside being bonded to the protein via C_PEB_–S_Cys_ covalent bond forms water-mediated H-bonds to the C-ring propionic group and to Glu60. These interactions help to hold the pigment’s ring D in place and in its particular orientation.

The earlier structure of PE from *Phormidium* sp. A09DM at pH 8.5 (PDB ID: 5aqd; Kumar et al. 2016) showed similar structural differences for the pigment PEB188β and its protein interactions between the ten β-subunits N to W, and two β-subunits M and X. However, in the structure at pH 5 all 12 β-subunits showed the same organization (like in β-subunits N to W in pH 8.5 structure) around PEB188β. Although, the authors of the earlier work (Kumar et al. 2016) concluded that the difference between these two crystal structures was connected with the pH difference of two crystallization conditions, they could not explain why all β-subunits in the pH 8.5 structure did not also show an identical organization of the PEB188β binding site. Examination of the crystal packing of our pH 7.5 1.14 Å structure shows that the crystal contacts with the neighboring hexamers are different for the PEB188β pigment, and for protein residues in this PEB’s vicinity, in the β-subunits M and X compared to those in β-subunits N to W. As described above, the PEB188β pigment and residue Arg148 in the β-subunits M and X make water-mediated contacts with residues Thr122 and Thr123 of the β-subunits W or N of the neighboring hexamers, respectively. We found that none of the PEB188β pigments in the ten β-subunits N to W form such crystal contacts in this region.

It was also found that Cα-carbon of the Arg148 in the β-subunits M and X is located at a distance of 5.09 Å from the NB atom, the nearest atom of the PEB188β pigment, while this distance for β-subunits N to W was found to be in a range of 4.74–4.89 Å. The difference in these distances, between M/X and N-to-W subunits, is in the range of 0.2–0.35 Å, so is about ten times larger than the estimated coordinate error 0.03 Å for the 1.14 Å structure. It is therefore highly significant and explains why π-stacking between Arg148 and PEB188β is not formed in β-subunits M and X. Remodelling of the side chain conformation for the Arg148 residue from β-subunit M has shown that when this side chain is made approximately parallel to the PEB’s C-ring to mimic the π-stacking to PEB188β, the shortest distance between the two planes is about 3.9–4.0 Å while an optimal π-stacking distance is expected to be ~3.6 Å. Distances in the range of 3.57–3.66 Å were found for all Arg148 π-stacking cases for the β-subunits N to W. These modelling trials reinforce significance of the differences between the M/X and N-to-W subunits and the effect they have on the π-stacking, which explains why efficient π-stacking is not formed in β-subunits M and X and the Arg148 side chain assumes different conformation. Examination of the pH 5 structure (PDB ID: 5fvb; Kumar et al. 2016) revealed that equivalent hexamer–hexamer contacts in the vicinity of the PEB188β pigment also exist for β-subunits M and X but the gap between the hexamers is a bit wider (by at least two layers of water). This explains why the PEB188β pigment structures in β-subunits M and X are not significantly different from those in β-subunits N to W at pH 5 but are different at pHs 7.5 and 8.5. This type of detailed discussion was not possible for the previous crystal structures of PE from *Phormidium* sp. A09DM due to their limited resolution.

All five PEB pigments are involved in multiple interactions with the surrounding apo-proteins. These interactions can be divided into three groups. The first group includes the C_PEB_–S_Cys_ covalent bond to the A-ring of the PEB molecule (or two such covalent bonds to rings A and D in case of PEB188β) and an aspartate residue, which forms two H-bonds to the pigment’s NB and NC atoms of its rings B and C, respectively (in case of PEB167α pigment this interaction is mediated by a water molecule). These interactions anchor the PEB molecules in a specific place within the protein scaffold. The second group of interactions includes a variety of H-bonds to PEB B- and C-ring propionic acid groups that fix the orientation of these two rings of PEB so that the conjugated system is well maintained. This orientation of the B- and C-rings is additionally stabilized in some cases by the π-stacking with key arginine residues. The third group of interactions involves a set of H-bonds that control deviation of the PEB A-ring relative to the axis defined by B- and C-rings. These three groups of interactions are most important for controlling the spectroscopic and energy transfer properties of the pigment molecules. There are also some H-bond interactions that control the orientation of PEB’s ring D relative to the axis of the B- and C-rings but these are less important functionally since ring D is not in conjugation with rings B and C. Table 2 summarizes all the interactions of PEB chromophores with the protein PE from *Phormidium* sp. A09DM discussed above and provides information about the conservation of these residues among available PE sequences from diverse cyanobacterial species. Total 37 orthologous sequences to PE-α and 34 to PE-β have been mined from the NCBI database and used for this analysis (these sequences are listed in the Supplementary Material Fig. S2). All of the interacting residues, except for the Arg148 from the insertion 146–152 in the β-subunits, are strongly conserved. The microenvironments of the PEB chromophores in the PE are remarkably highly conserved.

**Table 2:** Conservation of PE residues interacting with the PEB pigment molecules. Total of 37 orthologous sequences for PE-α and 34 for PE-β from the NCBI database were taken into consideration to analyse conservation (see Supplementary Material Fig. S2 for more details); (A) details of covalent bonds C_PEB_–S_Cys_ for each of PEB chromophores and conservation of the involved Cys amino acids; ‘x’ stands for non-conserved position; (B) list of H-bonds involved in chromophore–protein interactions and conservation of the involved amino acids; ‘x’ stands for non-conserved position

PEB, atom		Cys residue, atom		Conserved motif
(A)				
PEB166α	Ring A, CAA	Cys82	SG	81-K**C**xRD-85
PEB167α	Ring A, CAA	Cys139	SG	137-Rx**C**-139
PEB186β	Ring A, CAA	Cys80	SG	78-AA**C**L-81
PEB187β	Ring A, CAA	Cys165	SG	164-(D/R)**C**(A/S)-166
PEB188β	Ring A, CAA	Cys48	SG	45-NAS**C**-48
PEB188β	Ring D, CAD	Cys59	SG	58-I**C**E-60

Chromophore		Protein		Conserved motif (if conserved)
PEB	Atom	Residue	Atom	
(B)				
PEB166α	Ring A, NA	Ala72	O	70-GE**A**-72
	Ring C, O1C	Lys81	NZ	81-**K**CxRD-85
	Ring C, O2C	Arg84	NH1	81-KCx**R**D-85
	Ring C, O2C		NH2	
	Ring B, NB	Asp85	OD2	81-KCxR**D**-85
	Ring C, NC		OD2	
PEB167α	Ring B, O1B	Arg137	NH2	135-Rx**R**-137
	Ring B, O2B		NH1	
	Ring C, O1C	Arg142	NH2	141-P**R**D-143
	Ring C, O2C		NH1	
PEB186β	Ring A, NA	MeN70	OD1	68-GG**N**CYP-73
	Ring B, O2B	Arg75	NH1	75-**R**RMA-78
	Ring B, O1B	Arg76	NH2	75-R**R**MA-78
	Ring B, O1B		NE	
	Ring C, O2C	Arg82	NH1	80-CL**R**D-83
	Ring C, O2C		NH2	
	Ring B, NB	Asp83	OD2	80-CLR**D**-83
	Ring C, NC		OD2	
PEB187β	Ring D, OD	Gln28	NE2	27-(V/I)**Q**G-29
	Ring D, ND	Asn33	OD1	32-(G/A)**N**(R/K)R-35
	Ring B, O1B	Arg34	NE	32-(G/A)N(**R**/K)R-35
	Ring B, O2B		NH2	
	Ring B, NB	Asp37	OD2	33-N(R/K)RL**D**A-38
	Ring C, NC		OD2	
	Ring A, NA	Pro159	O	Main chain carbonyl-*O* conserved irrespactable to primary sequence
	Ring A, OA	Val161	O	Main chain carbonyl-O conserved irrespactable to primary sequence
PEB188β	Ring B, NB	Asp52	OD2	50-VS**D**A-53
	Ring C, NC		OD2	
	Ring C, O1C	Agr127	NH1	127-**R**AV-129
	Ring B, O1B	Ser145	N	144-x(**S**/**T)**x-146
	Ring B, O2B		OG	
	Ring C, ND	Arg148	NH1	146–152 loop insertion, not conserved

### Conformation of the protein loop near the PEB188β pigment

In PE from *Phormidium* sp. A09DM there is an insertion of 7 amino acids in the β-subunits relative to the sequence of most PEs (whose crystal structures are available), which forms an extension at the end of the large loop (that is composed of residues 143–161) in vicinity of the PEB188β pigment. This insert wraps over the top of the PEB188β on its solvent phase side and contributes the important Arg148 residue whose role was described above. The NCBI database of 34 sequences of β-subunits of PE from different species of cyanobacteria that contain this insertion loop (of length of 7–9 amino acids) has been analysed. A multiple sequence alignment table for these sequences is shown in Fig. S2 in the Supplementary Material. Although there is a certain degree of sequence conservation in this insertion there are no positions where the type of amino acid is completely conserved. Moreover, an X-ray crystal structure for PE containing this insertion is only available for the species of cyanobacteria used here.

**Fig. 4:**
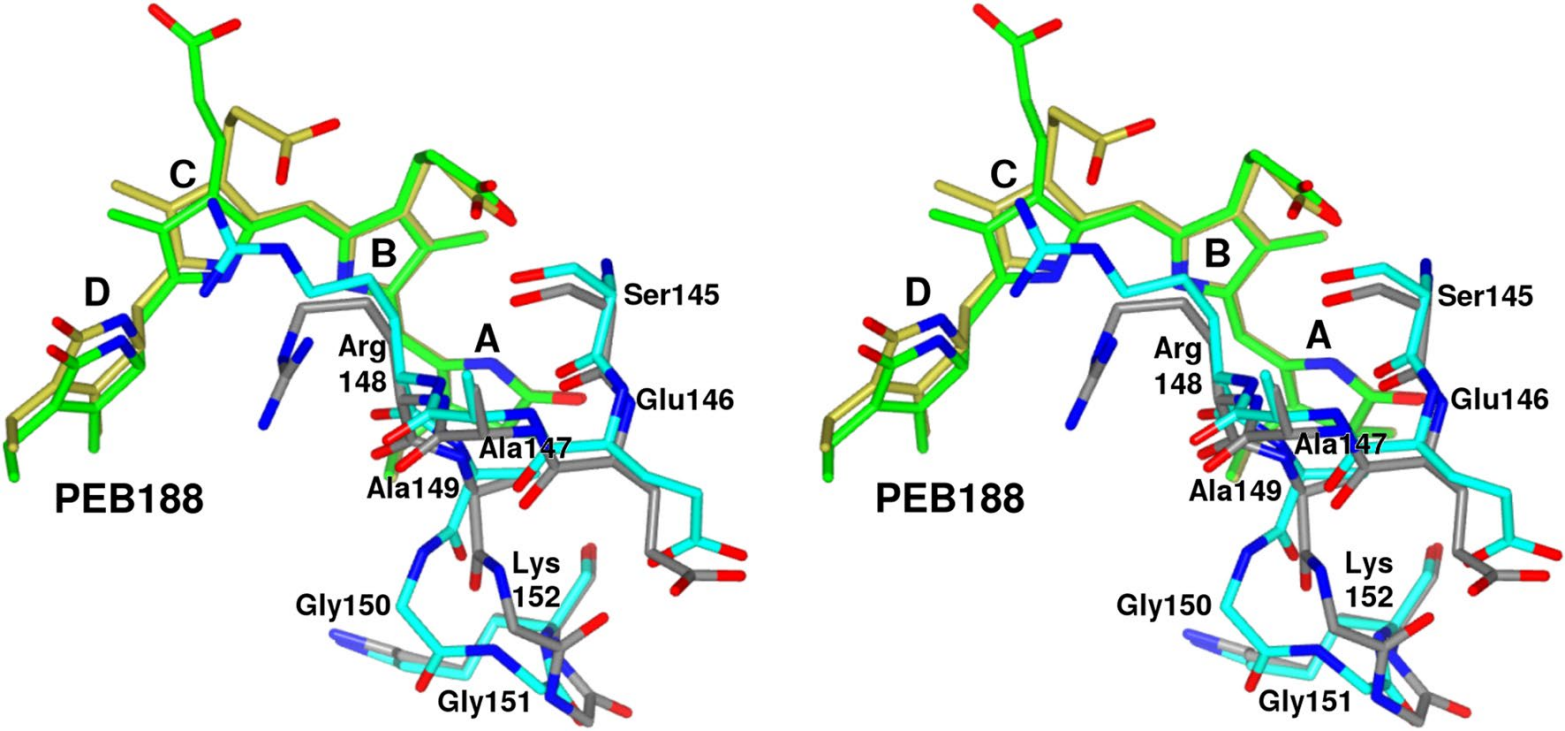
Stereo view of superimposed models of the inserted loop Glu146-Lys152 partially overlapping chromophore PEB188 in β-subunit N (cyan protein chain and green chromophore) and in β-subunit M (grey protein chain and golden chromophore). The conformation of this loop in nine β-subunits O to W is the same as in β-subunit N, while the conformation in β-subunit X is the same as in M. This figure was prepared using *CCP*4mg (McNicholas et al. 2011)

In the 1.14 Å structure of PE from *Phormidium* sp. A09DM reported here an interesting curiosity about the conformation of this insertion was found. Namely, the 149-152 fragment of this insertion was found to be in two different main chain conformations in the crystal structure. One conformation is seen in ten β-subunits, N to W, and is associated with the Arg148 π-stacking with the PEB188β molecule. The alternative second conformation is seen in β-subunits M and X, where the Arg148 does not form the π-stacking interaction with the PEB188β. These two main chain conformations of the loop together with the associated PEB188β molecules are shown overlaid in Fig. 4. An obvious conclusion would be to suggest that these two conformations are correlated with the type of interactions Arg148 has with the chromophore PEB188β and are a consequence of these interactions. However, another of the two PE structures we are reporting here, the one refined based on the 1.38 Å data obtained for the crystal grown in the mix of carboxylic acids (Morpheus screen G8 condition) presents another case. In that structure, the conformation of the 146–152 fragment of the loop in β-subunits M and X is the same as in the 1.14 Å structure, but the situation in β-subunits N to W is different. The electron density maps for this structure suggested presence of both conformations, therefore the fragment 146–152 of the loop in these ten β-subunits was modelled using a mixture of both conformations observed in 1.14 Å structure in the ratio of approximately 60:40%, i.e. with a slightly higher contribution of the conformation present in β-subunits M and X. Confusingly, at the same time the Arg148 π-stacking interactions with the PEB188β pigment are identical to those seen in 1.14 Å structure, i.e. different in β-subunits M/X compared to the situation in subunits N to W. The reason for this complication is not clear but probably reflects the differences in the crystallization conditions used to produce these two crystal forms. Examination of the 1.93 Å (pH 5, 5fvb) and 2.12 Å (pH 8.5, 5aqd) structures (Kumar et al. 2016) revealed still another situation, namely all the β-subunits in both structures were modelled with one loop conformation (the one seen in our β-subunits M and X). However, there were also residual electron densities in the vicinity of this loop that suggested some partial presence of the alternative conformation as well, but modelling of these alternative conformations was not attempted at these lower resolutions. These findings reiterate the unquestionable value of being able to examine crystal structures at the atomic resolution.

### Additional structural features

**Fig. 5:**
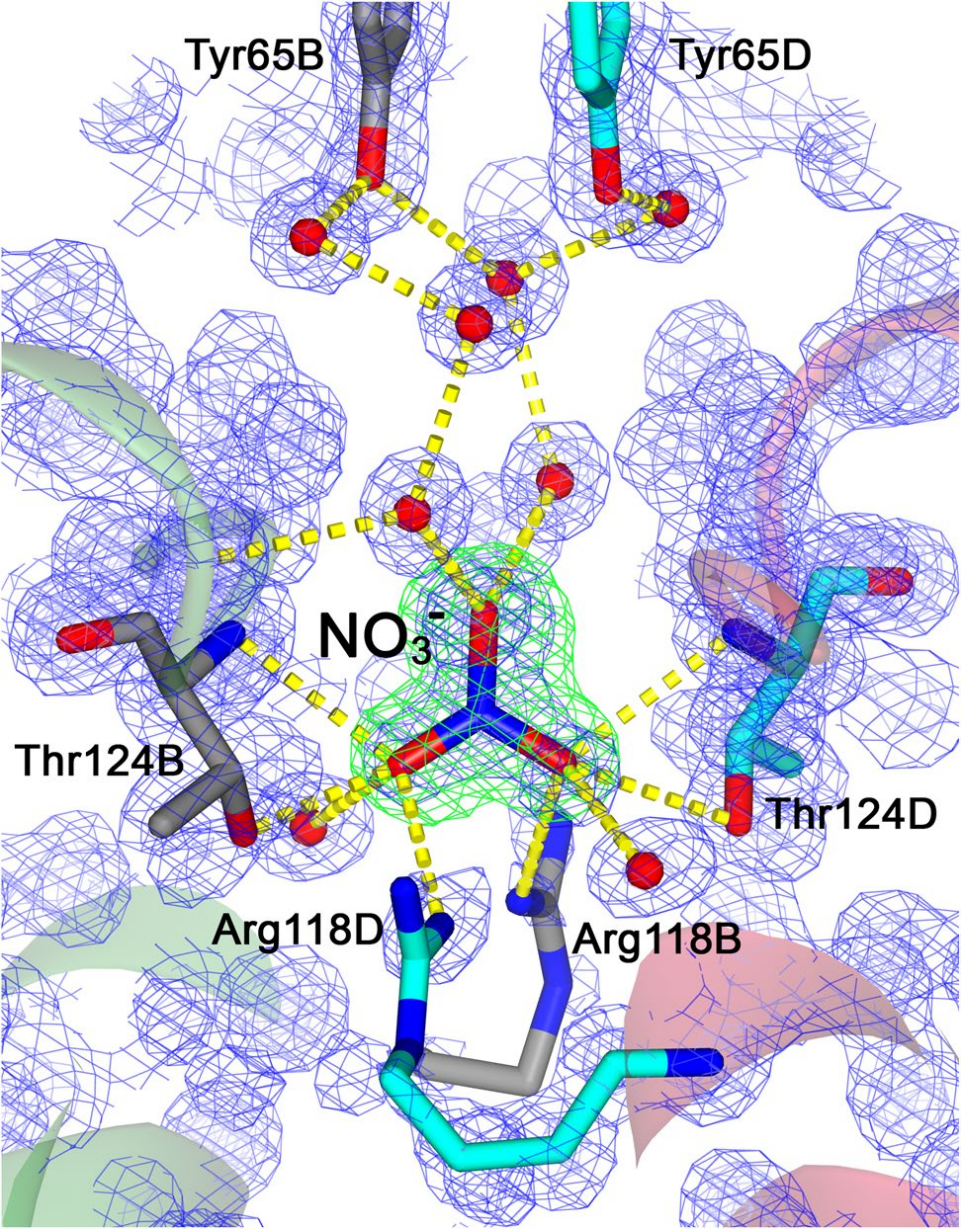
A representative example of one of the six nitrate (NO_3_^−^) anions that bridge four PE trimers by H-bonding network to form two hexamers {[(αβ)_3_]_2_}. The NO_3_^−^ anion together with several water molecules make an ‘H-bridge’ by involvement of Thr124, Arg118 and Tyr65 residues from α-subunits B (cyan) and D (grey) of two trimers. Hydrogen bonds are presented as yellow dashed lines. Water molecules are shown as red spheres. Electron density map *2Fo-Fc* and the omit difference Fourier map (for the NO_3_^−^ anion) are shown as blue and green meshes at contour levels 1.5 sigma and 4.0 sigma, respectively. This figure was prepared using *CCP*4mg (McNicholas et al. 2011)

In our 1.14 Å structure of PE (crystals grown in Morpheus C8 conditions) α-subunits of two trimers are bridged by three well-ordered nitrate anions (NO_3_^−^) each forming contacts with two threonine residues (Thr124) from two α-subunits across each boundary between [(αβ)_3_] trimers. The details of this interaction are illustrated in Fig. 5. Apart from the H-bonds to the threonine residues the nitrate anion is H-bonded to Arg118 residues and through well-ordered water molecules to Tyr65 residues at the trimer: trimer interface. It is worth noting that in this case nitrate ions were present in the crystallization conditions containing the NPS mix of additives (NPS). Interestingly, in our 1.38 Å structure, where the crystals were grown in the absence of nitrates (in Morpheus G8 conditions, mix of additives: carboxylic acids) the equivalent site to that shown in Fig. 5 is occupied by ordered water molecules alone. This suggests that the binding of nitrate ions in this site is not essential for the stabilization of the hexamer. In the previous study by Kumar et al. (2016), it was shown that this site in the crystal grown at pH 8.5 (PDB ID: 5aqd) could also be occupied by a sulphate anion (where sulphate ions were present in their crystallization conditions).

**Fig. 6:**
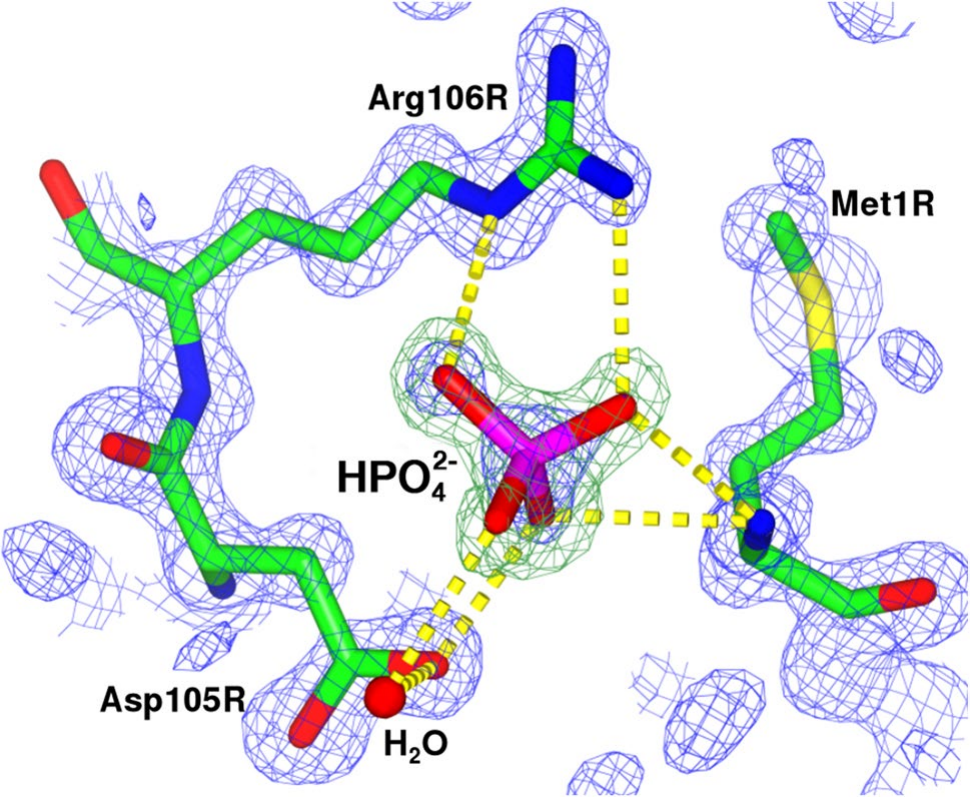
A representative of one of the twelve hydrogen phosphate (HPO_4_^2−^) ions interacting with β-subunits at the N-termini by H-bonding to Met1, Asp105 and Arg106 residues. Hydrogen bonds are presented as yellow dashed lines. A water molecule is shown as the red sphere. Electron density map *2Fo–Fc* and the omit difference Fourier map (for the HPO_4_^2−^ ion) are shown as blue and green meshes at contour levels 1.5 sigma and 3.0 sigma, respectively. This figure was prepared using CCP4MG (McNicholas et al. 2011)

Crystallization conditions used here that produced the 1.14 Å PE structure also contained phosphate ions. As shown in Fig. 6 phosphate anions, which were modelled as HPO_4_^2−^ ions for the crystallization pH of 7.5, were found H-bonded to residues Met1, Asp105 and Arg106 at the N-terminal region of each β-subunit. Another more tentative binding site for phosphate was also identified in the α-subunit forming H-bonds with residues Gly112, Arg114 and Glu115. Both these sites are close to the central cavity inside each trimer. These sites in the 1.38 Å structure were occupied by water molecules only.

### Fitting the absorption spectrum of PE: implications for energy transfer

**Fig. 7:**
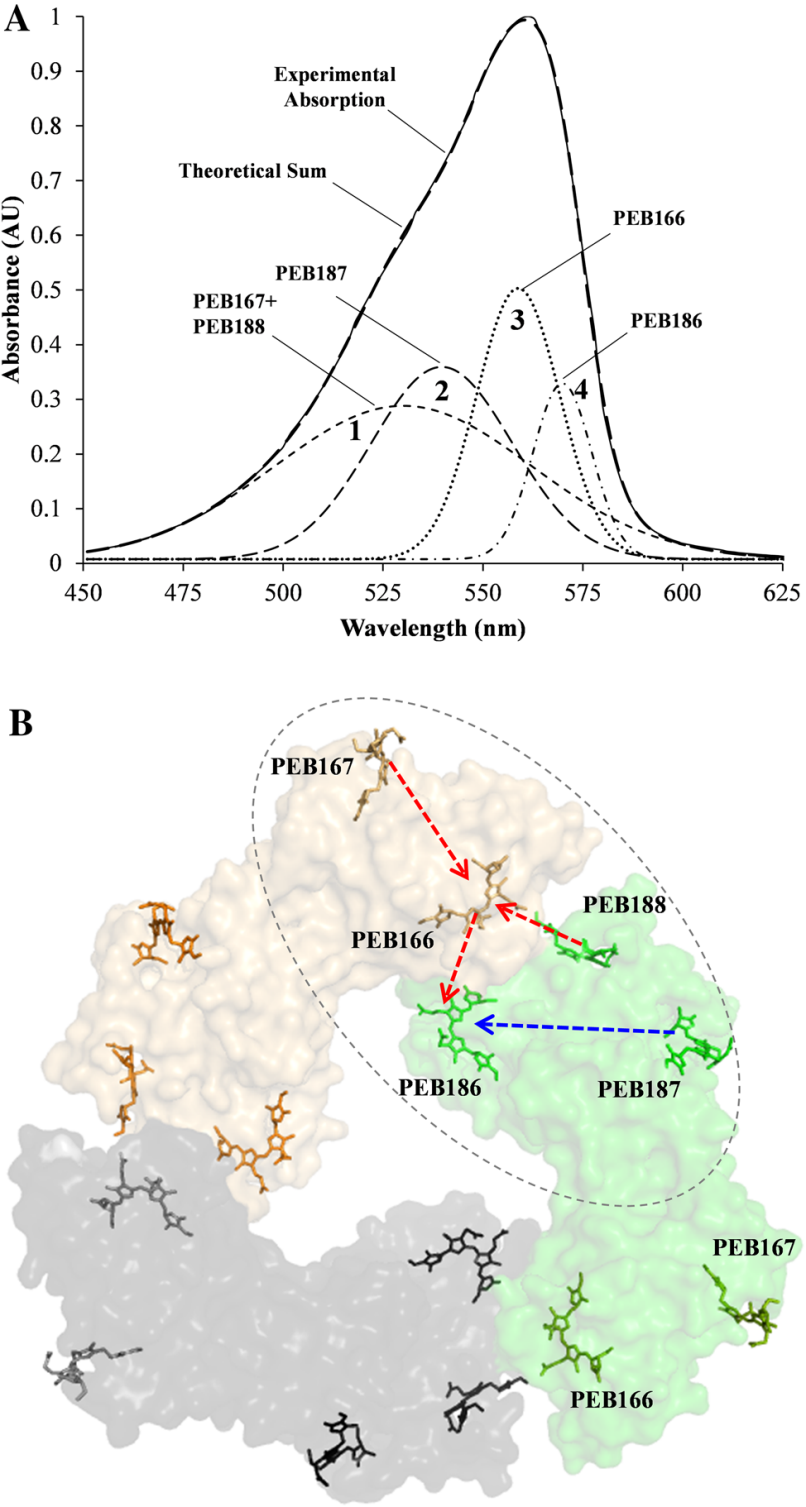
Deconvolution of the PE absorption spectrum and tentative energy transfer pathways **a** deconvolution of the PE absorption spectrum into four Gaussian decomposition components designated as dashed-line peaks *1* to *4* associated with the named pigment molecules. Intact line represents the experimental steady-state absorption profile of PE. The quality of the deconvolution fit is shown by the bold dashed line that represents the addition of the four deconvoluted Gaussian components, **b** the spatial arrangement of chromophores in the PE trimer and the two proposed energy transfer pathways (represented by the red and blue dashed lines)

The CD spectrum of PE was recorded in 400–600 nm region. This spectrum just shows a featureless broad positive signal throughout this region (see Fig. S3 in the Supplementary Material). More useful information was obtained by deconvolution of the PE absorption spectrum. The best fit for this deconvolution was obtained with four separate Gaussian peaks (Fig. 7a). These peaks have absorption maxima (λ_max_) at 530, 540, 559, 570 nm, respectively. Based on the A–B-ring planarity data (Table S2) and as described in Doust et al. (2004) and Choubeh et al. (2017), these peaks were tentatively assigned to individual chromophores, i.e. Peak 1 (530 nm) was assigned to high-energy PEB167 + PEB188 (the contributions of these two pigments could not be further resolved), Peak 2 (540 nm) to PEB187, Peak 3 (559 nm) to PEB166 and Peak 4 (570 nm) to low-energy absorbing PEB186 (Fig. 7a). Thus, PEB167 and PEB188 are positioned at blue end of the absorption band, PEB187 occupies the green region and PEB166 and PEB186 are at red end. The spatial arrangement of chromophores within PE trimer (Fig. 7b) suggests that the α- and β-chromophores within an αβ-monomer are too far apart for energy transfer, but upon formation of the trimer, the α-subunit chromophores of one αβ-monomer come into closer contact with the β-subunit chromophores of the neighboring αβ-monomer as indicated by the dashed oval in Fig. 7b. In this arrangement, the high-energy absorbing chromophores PEB167, PEB187 and PEB188 (Peak 1 and 2 in Fig. 7a) are located on the periphery, whereas low-energy absorbing chromophores PEB166 and PEB186 (Peak 3 and 4 in Fig. 7a) are located in middle layer and core, respectively. Using this information it is possible to suggest two pathways for energy transfer from the outwardly positioned high-energy chromophores to the inner low-energy chromophores; first PEB167 + PEB188 → PEB166 → PEB186 (dashed red arrows in Fig. 7b) and second PEB187→PEB186 (dashed blue arrow in Fig. 7b).

## Electronic supplementary material

Below is the link to the electronic supplementary material.

Supplementary material 1 (DOCX 17933 KB)

## Abbreviations

MPD: (4S)-2-methyl-2,4-pentanediol
APC: Allophycocyanin
PE: C-phycoerythrin
DLS: Diamond Light Source
MeN: Methylated Asn, γ-*N*-methylasparagine
NPS: Nitrate Phosphate Sulphate
OD: Optical density
PC: Phycocyanin
PEB: Phycoerythrobilin
PEG-1K: Polyethylene glycol 1000
