# Supplementary material for: An improved crystal structure of C-phycoerythrin from the marine cyanobacterium Phormidium sp. A09DM
Source: Photosynth Res. 2017 Sep 16;135(1):65–78. doi: 10.1007/s11120-017-0443-2 (PMC5783998; doi:10.1007/s11120-017-0443-2)

***Supplementary Material for the manuscript:***

**An improved crystal structure of C-phycoerythrin from the marine cyanobacterium *Phormidium* sp. A09DM**

by

Ravi R. Sonani^1#^, Aleksander W. Roszak^2#^*, Claire Ortmann de Percin Northumberland^2^, Datta Madamwar^1^*, Richard J. Cogdell^2^

**Table S1**. Optimized crystallization parameters for PE

|  | **Condition C8, Morpheus screen** | **Condition G8, Morpheus screen** |
| --- | --- | --- |
| Crystallization plate type | 24-well seating drop plate (Cryschem, NBS Biologicals). | 24-well seating drop plate (Cryschem, NBS Biologicals) |
| Temperature (K) | 294 | 294 |
| Protein concentration | 10 mg mL^-1^ | 10 mg mL^-1^ |
| Buffer composition of protein solution | 1.0M Sodium HEPES + MOPS (acid) (pH 7.5) (Buffer System 2 of Morpheus, Molecular Dimensions) | 1.0M Sodium HEPES + MOPS (acid) (pH 7.5) (Buffer System 2 of Morpheus, Molecular Dimensions) |
| Composition of reservoir solution | **Precipitants**  13 % MPD (racemic),  13 % PEG 1000,  13 % PEG 3350  **Salt/Ligands**  30 mM NaNO_3_,  30 mM Na_2_HPO_4_,  30 mM (NH_4_)_2_SO_4_ | **Precipitants**  13 % MPD (racemic),  13 % PEG 1000,  13 % PEG 3350  **Salt/Ligands**  20 mM Sodium formate,  20 mM Ammonium acetate,  20 mM Sodium citrate tribasic dehydrate,  20 mM Sodium potassium tartrate tetrahydrate,  20 mM Sodium oxamate |
| Volume and ratio of drop | Drop volume 10 µL with protein and reservoir in 1:1 ratio | Drop volume 10 µL with protein and reservoir in 1:1 ratio |
| Volume of reservoir | 600 µL | 600 µL |

| **Chromophore** | **A/M** | **B/N** | **C/O** | **D/P** | **E/Q** | **F/R** | **G/S** | **H/T** | **I/U** | **J/V** | **K/W** | **L/X** | **Avg** | **STDev** |
| --- | --- | --- | --- | --- | --- | --- | --- | --- | --- | --- | --- | --- | --- | --- |
|  | **1** | **2** | **3** | **4** | **5** | **6** | **7** | **8** | **9** | **10** | **11** | **12** |  |  |
| **PEB166** | 24.29 | 25.78 | 26.26 | 25.33 | 26.65 | 25.19 | 26.83 | 24.03 | 26.65 | 26.02 | 24.15 | 25.9 | 25.59 | 0.999 |
| **PEB167** | 40.03 | 41.09 | 37.28 | 41.85 | 36.97 | 37.13 | 36.57 | 36.48 | 41.59 | 42.31 | 40.74 | 40.21 | 39.35 | 2.277 |
| **PEB186** | 20.4 | 22.21 | 20.69 | 22.34 | 21.34 | 22.26 | 22.06 | 22.63 | 21.46 | 20.28 | 19.85 | 22.08 | 21.47 | 0.944 |
| **PEB187** | 34.03 | 33.56 | 30.26 | 31.46 | 31.62 | 34.32 | 33.39 | 32.74 | 33.57 | 32.97 | 33.21 | 32.45 | 32.80 | 1.180 |
| **PEB188** | 42.54 | 43.8 | 43.7 | 43.72 | 41.14 | 42.27 | 43.4 | 41.18 | 43.4 | 42.33 | 42.04 | 41.62 | 42.60 | 0.990 |

**Table S2.** The angles between rings A and B for the five PEB molecules in twelve molecules in the *P*1 crystal unit cell for the pH 7.5 1.14 Å PE structure

**Fig. S1.** The 2-D LigPlot+ representation of the chromophore-protein interactions. The interactions of PEB167A (A), PEB187M (B), PEB186M (C), PEB166A (D), PEB188N (E), PEB188M (F) with surrounding residues in the range of 3.9 Å. Chromophore is represented by ball-stick model with raspberry bond colour. Residues forming covalent and H-bonds with the chromophore atoms are shown as ball-sticks model with yellow bond colour. Residues making hydrophobic contact(s) are represented by red colour (radiating) partial circle along with residue labels. H-bonds and covalent bonds C_PEB_-S_Cys_ are shown in dashed-blue and intact-green lines, respectively. Carbon, nitrogen, oxygen and water molecules are represented by black, blue, red and aqua colour balls, respectively. The figure was prepared using LigPlot+ suite (Laskowski RA and Swindells MB (2011) J Chem Inf Model 51: 2778-2786)

**Fig. S1A**

**
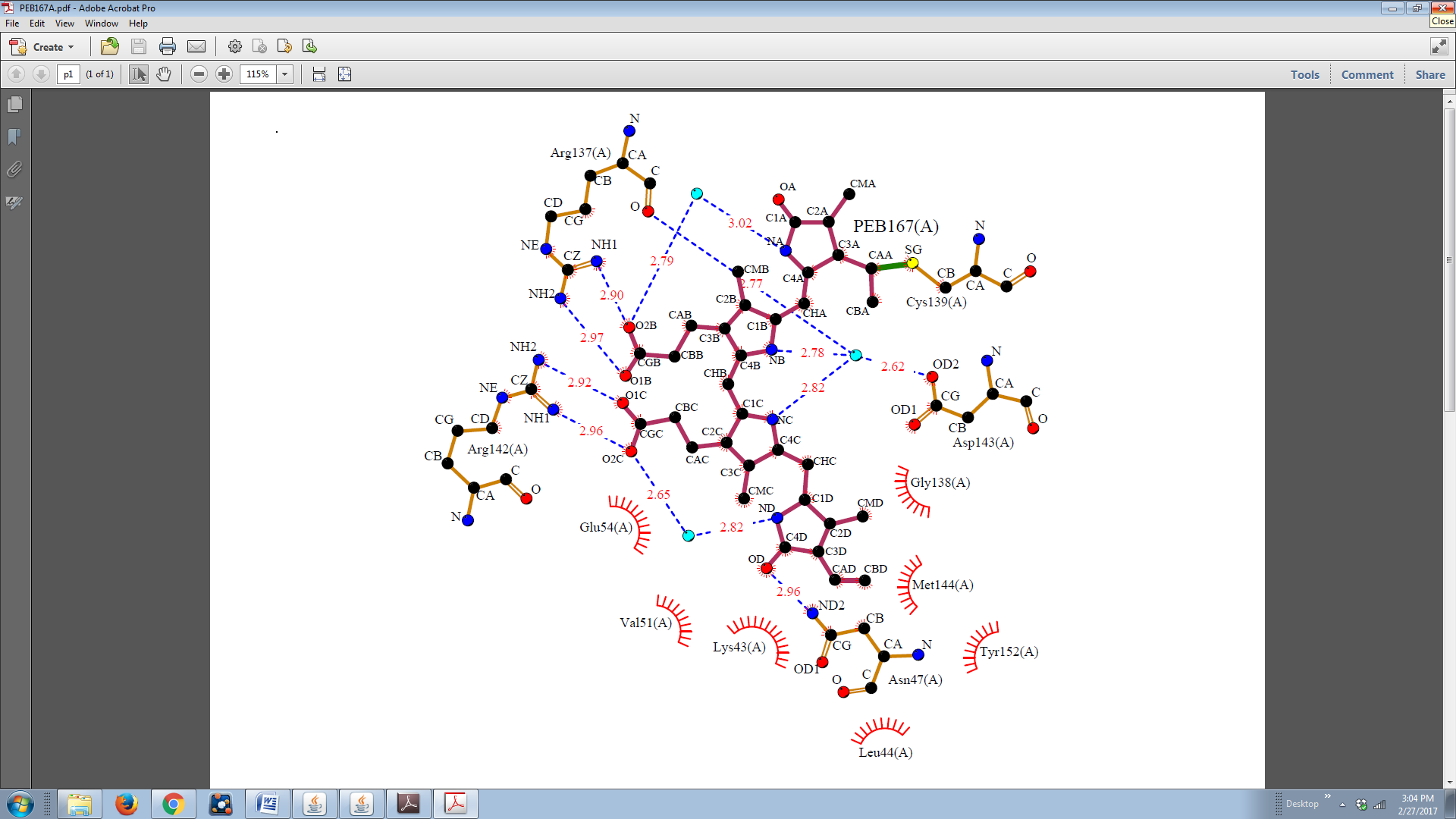
**

**
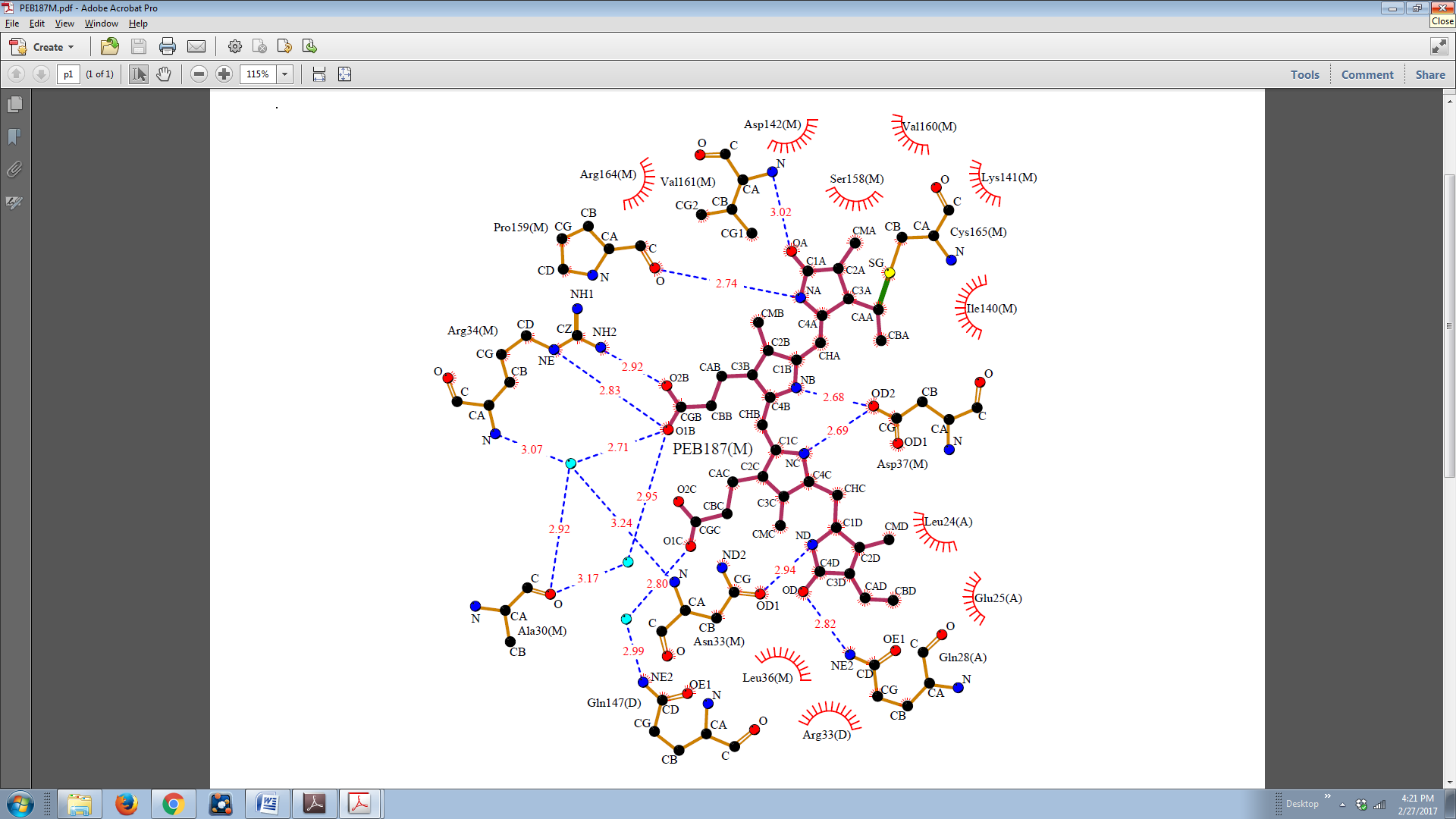
Fig. S1B**

**Fig. S1C**

**
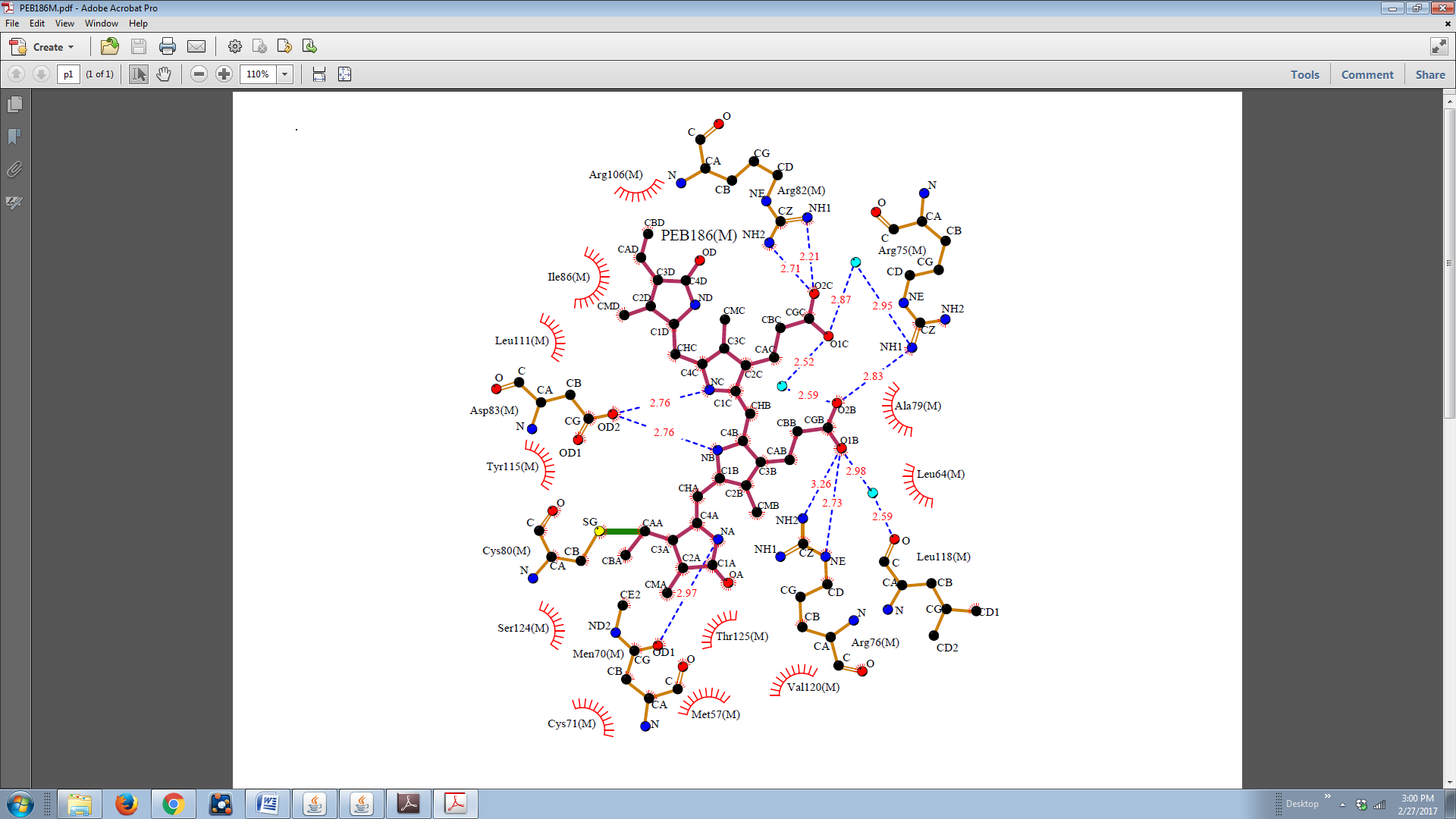
**

**Fig. S1D**

**
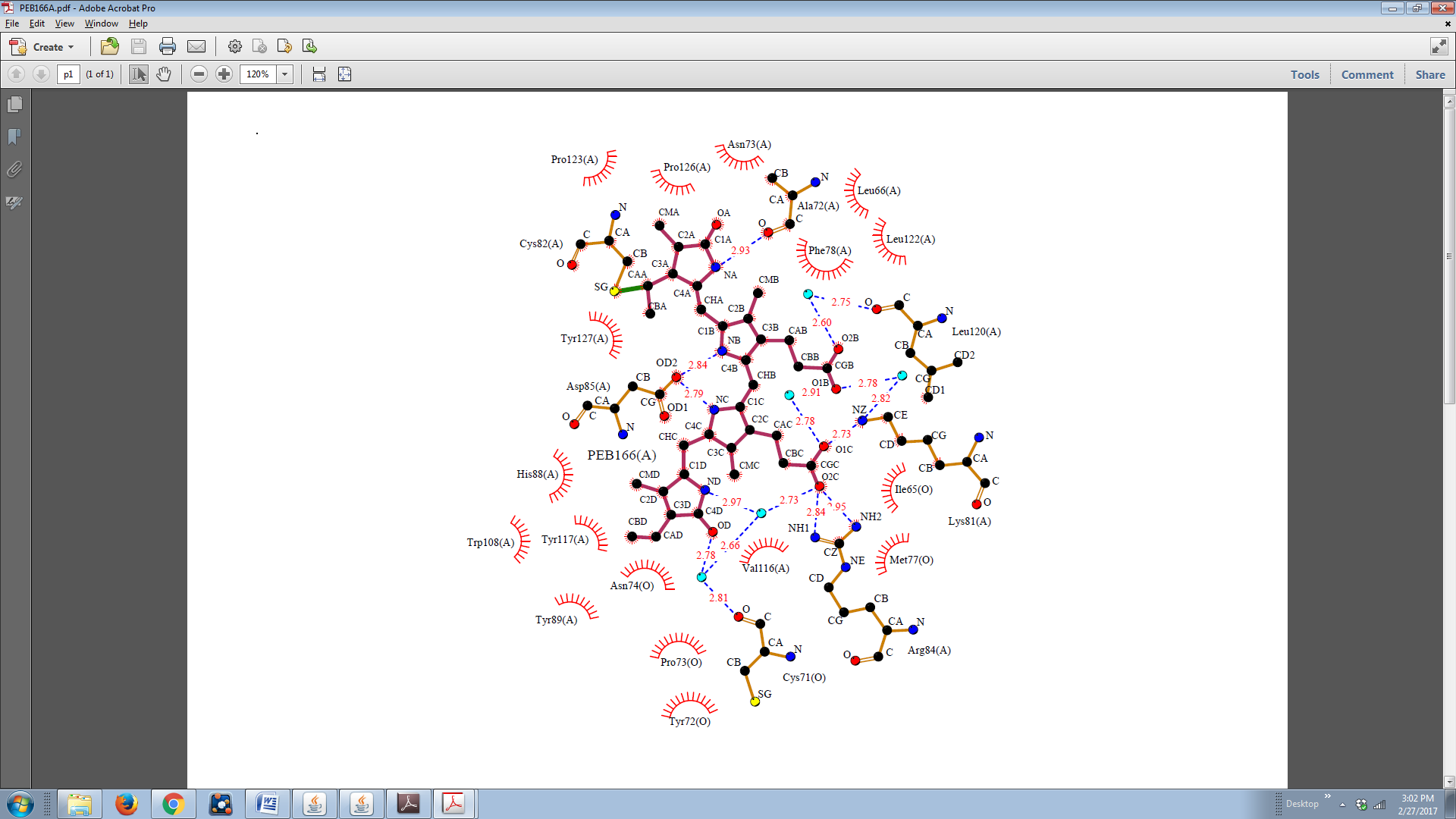
**

**Fig. S1E**

**
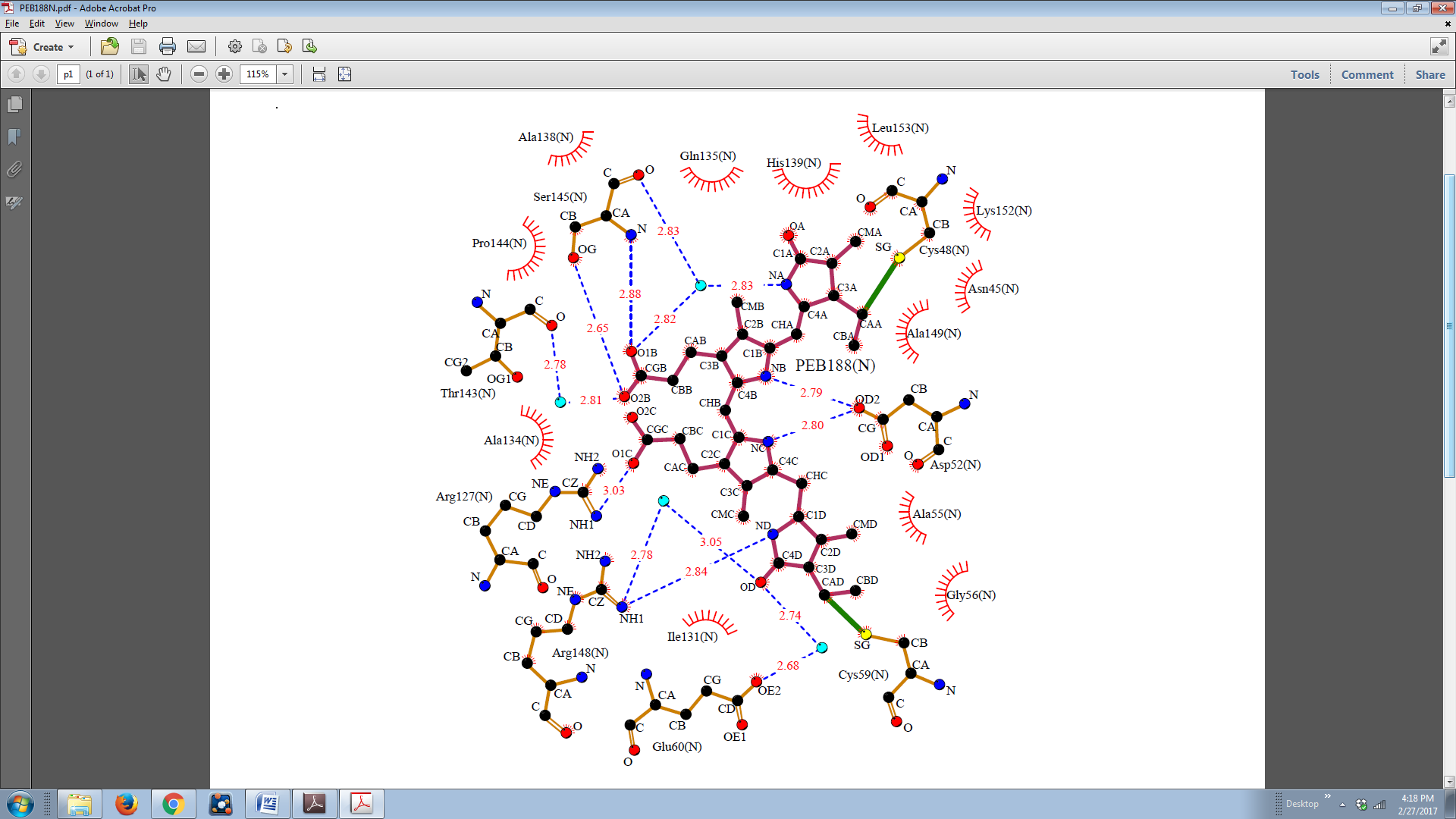
**

**Fig. S1F**

**
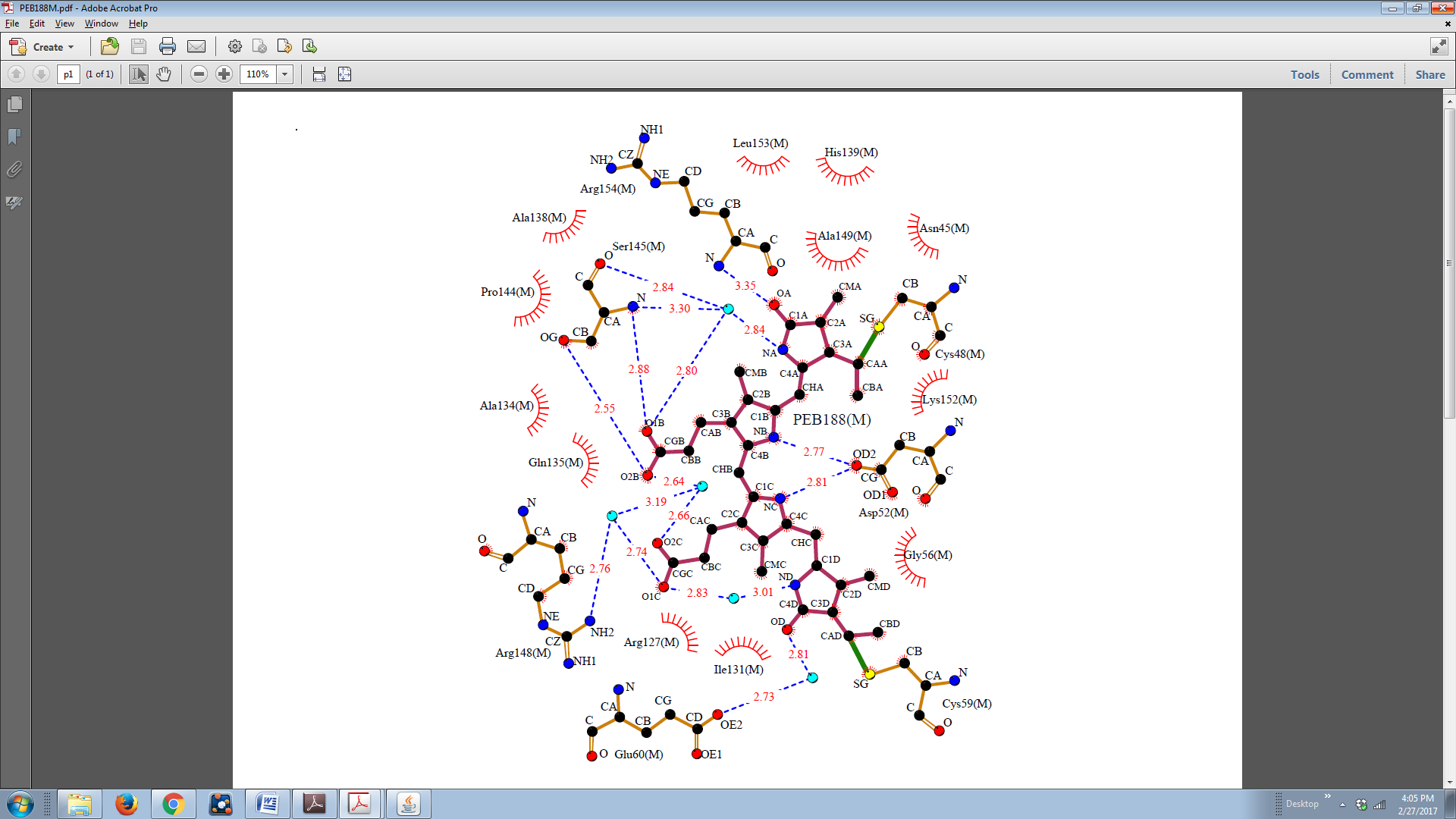
**

**Fig. S2**. Result of multiple sequence alignment (MAS) of orthologus protein sequences to PE-β (A) and PE-α subunit (B), retrieved from the NCBI. Highly and moderately conserved sequences are highlighted by red and yellow color, respectively. The MAS was performed using EsPript 3.0 (Robert X and Gouet P, (2014) Nucl Acids Res 42(W1): W320-W324)

**Fig. S2A**

**
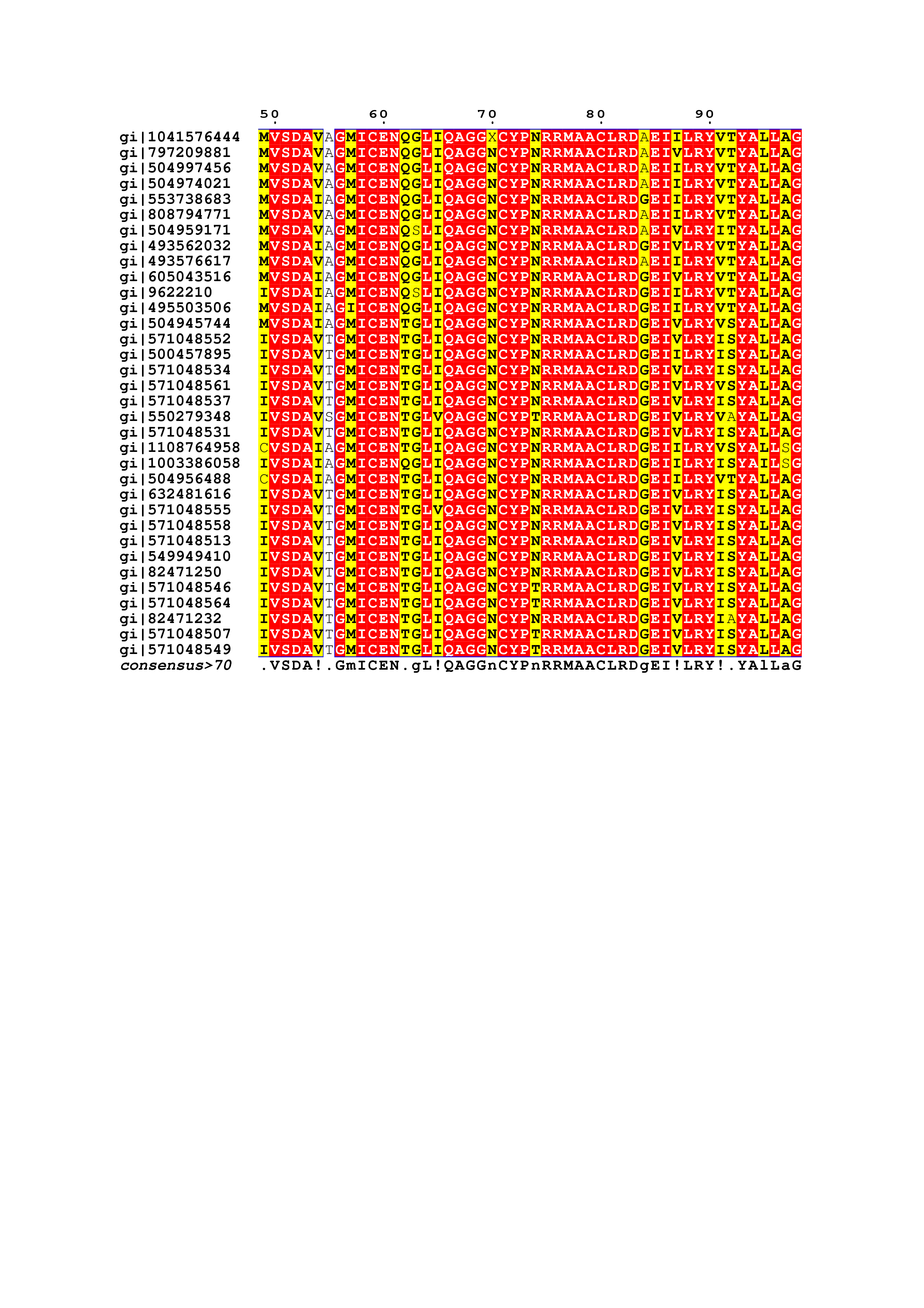

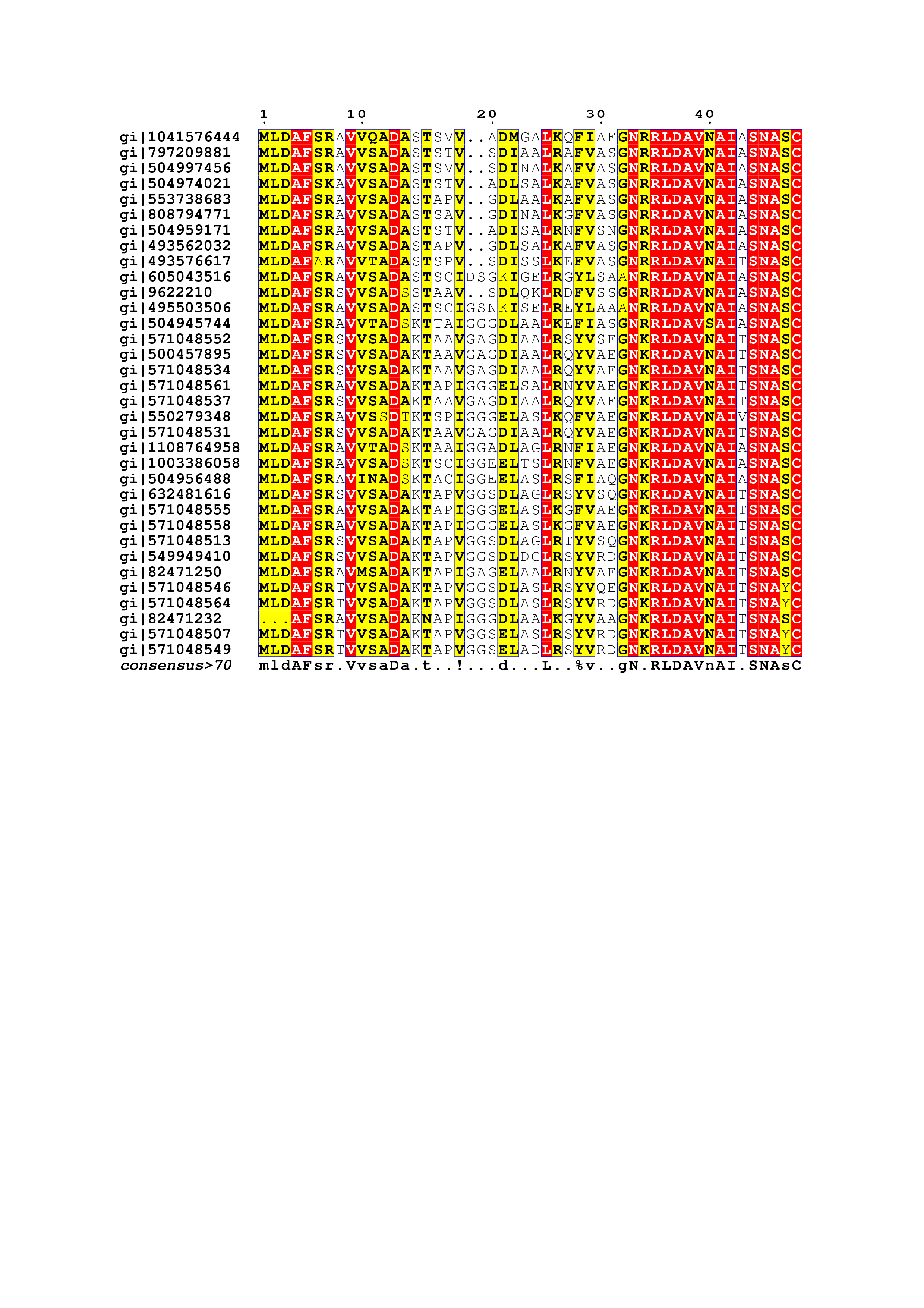
**

**Fig. S2A (Contd.)**

**
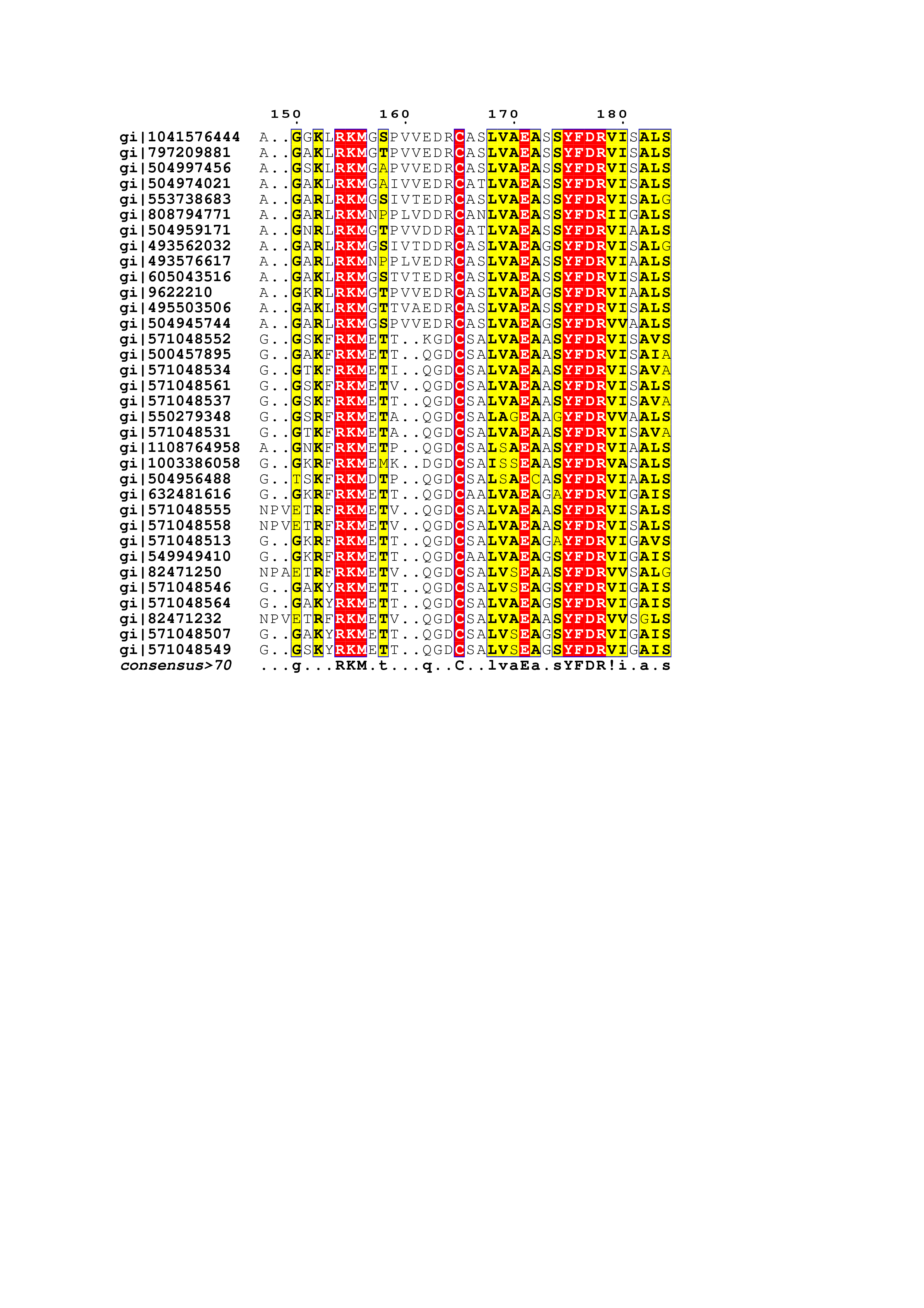

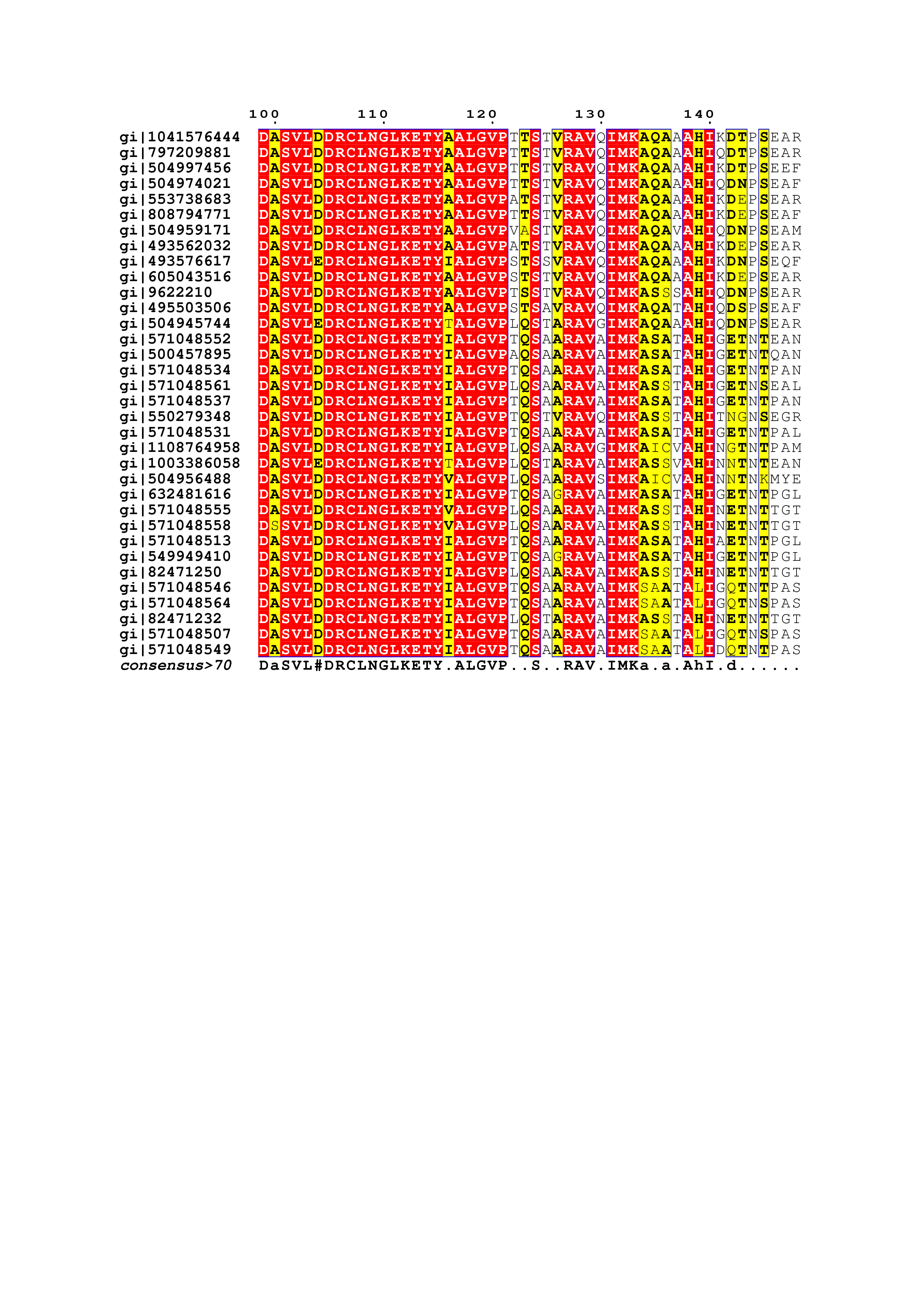
**

**Fig. S2B**


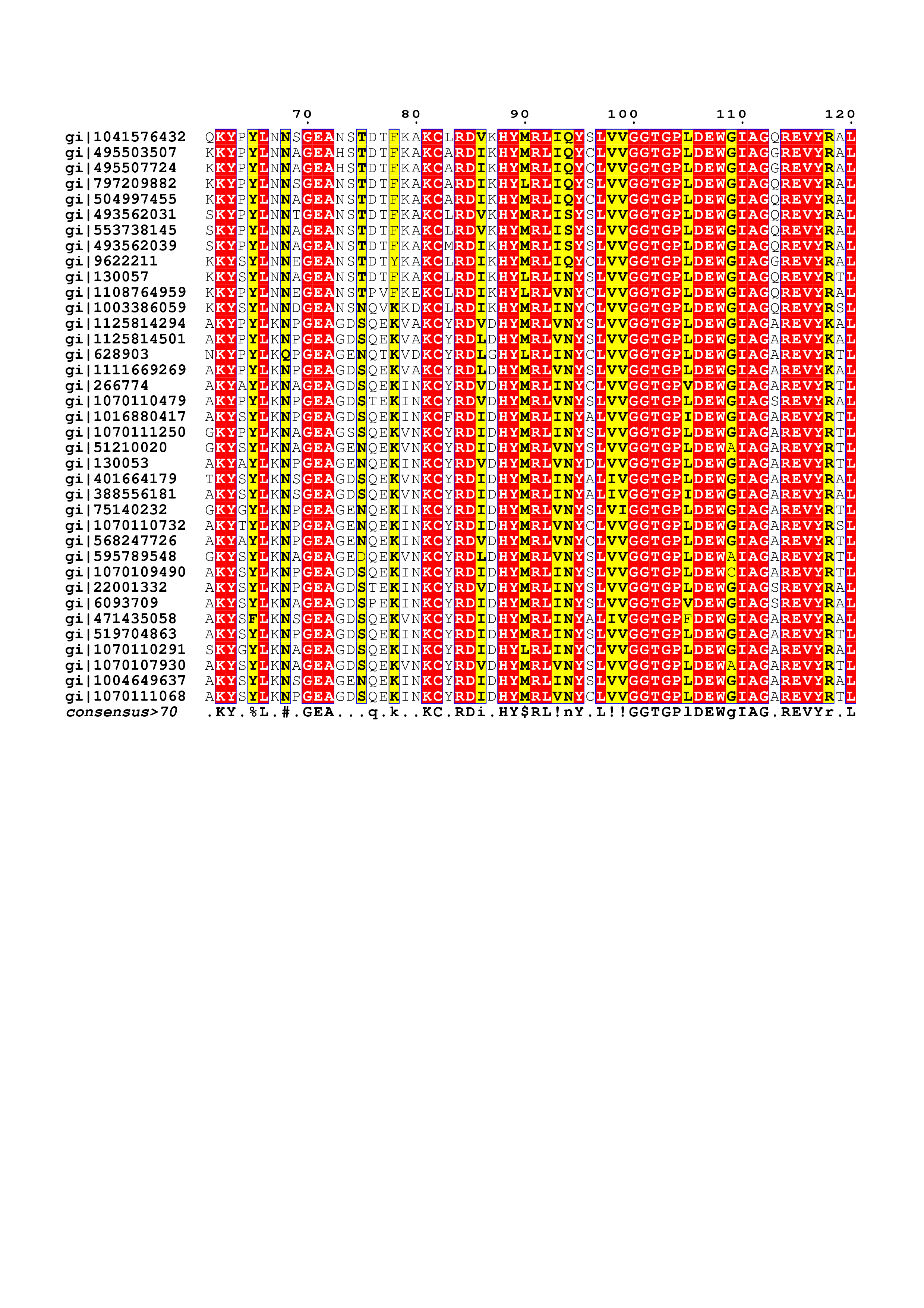

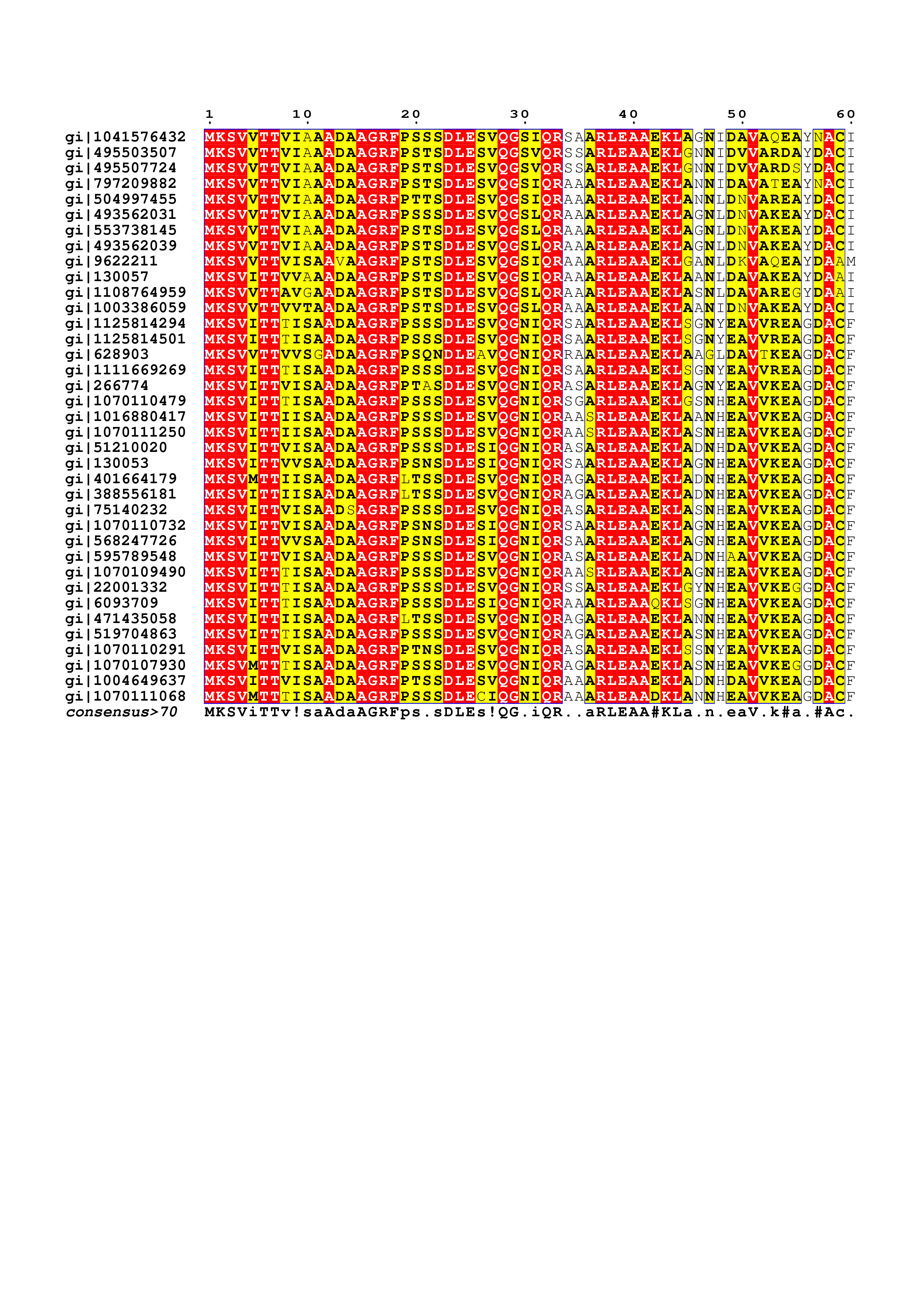


**
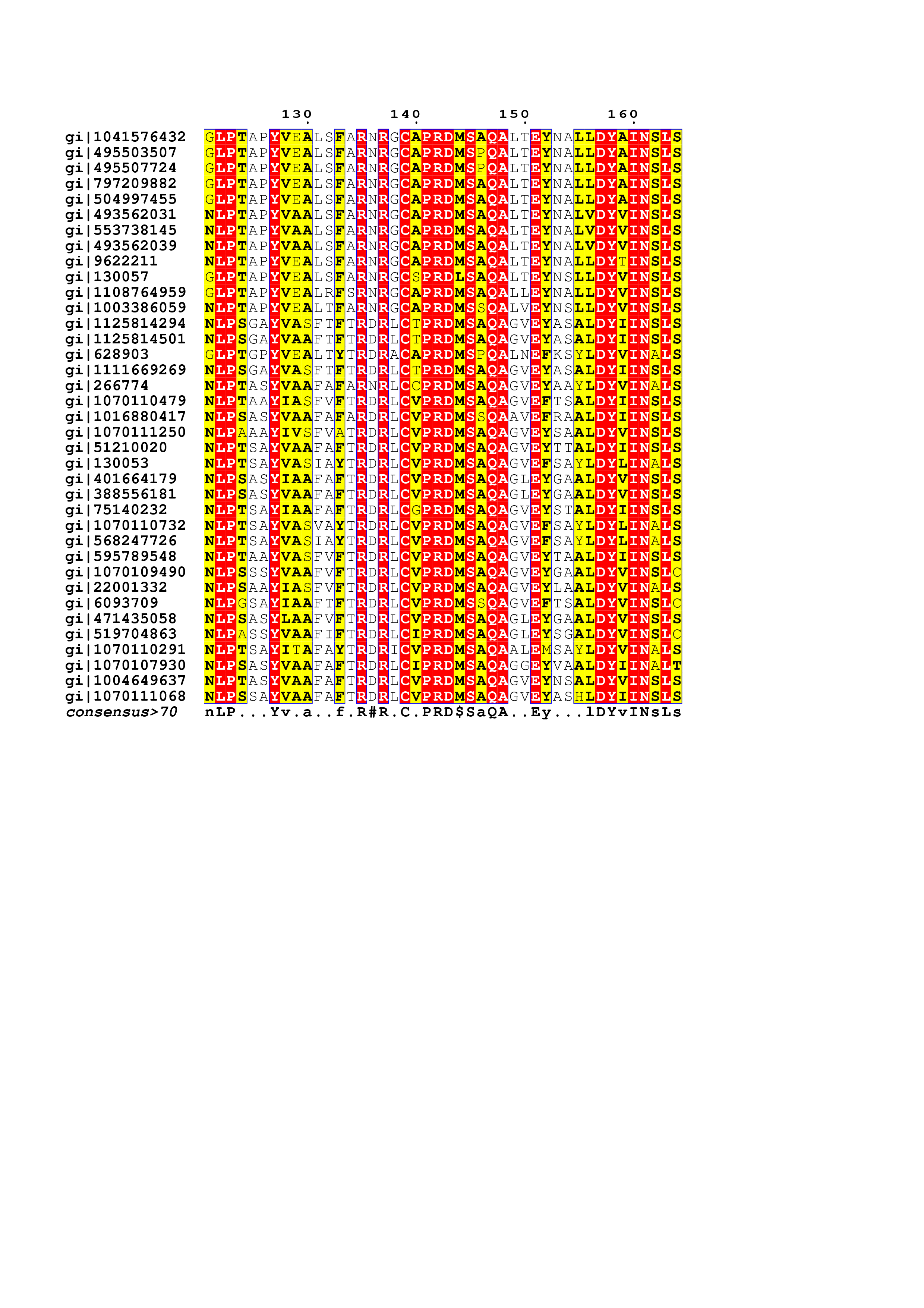
Fig. S2B (Contd.)**

**Fig. S3**. CD spectrum of PE from the marine cyanobacterium *Phormidium* sp. A09DM recorded at room temperature in the region of 400-600 nm. The PE sample was disolved in 20 mM Tris-buffer (pH 8.0) and had an absorption of 1 at the maximum of the absorption spectrum.


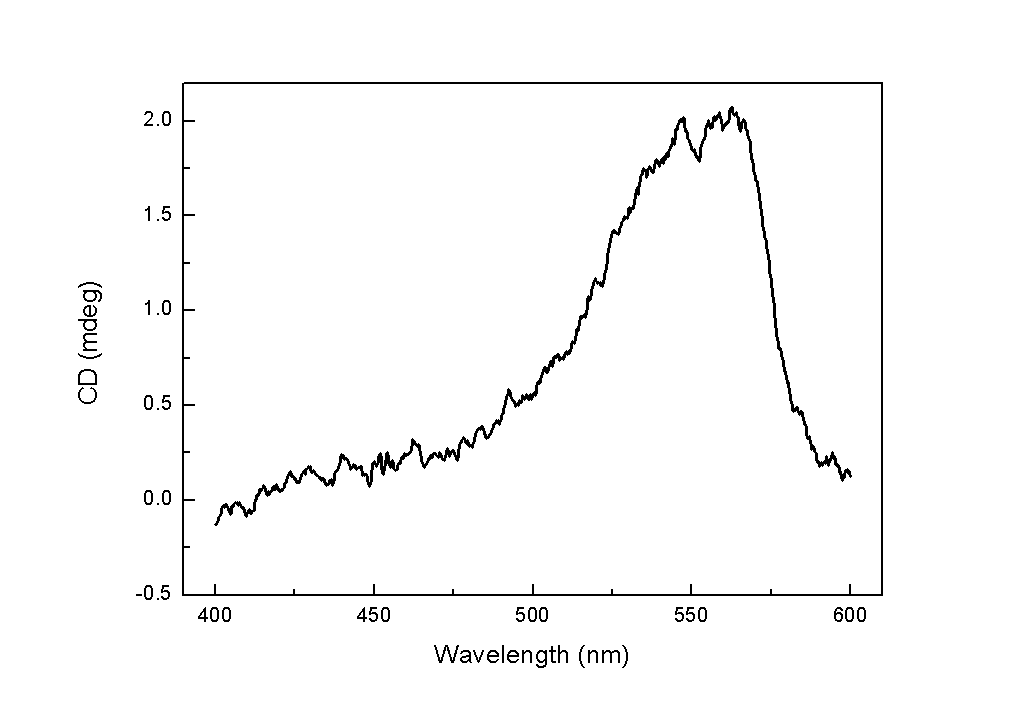

Supplement: Supplementary file 1 — Supplementary material 1 (DOCX 17933 KB) [file 11120_2017_443_MOESM1_ESM.docx]
